# Modelling capture efficiency of single-cell RNA-sequencing data improves inference of transcriptome-wide burst kinetics

**DOI:** 10.1093/bioinformatics/btad395

**Published:** 2023-06-24

**Authors:** Wenhao Tang, Andreas Christ Sølvsten Jørgensen, Samuel Marguerat, Philipp Thomas, Vahid Shahrezaei

**Affiliations:** Department of Mathematics, Imperial College London, London SW7 2BX, United Kingdom; Department of Mathematics, Imperial College London, London SW7 2BX, United Kingdom; I-X Centre for AI in Science, Imperial College London, White City Campus, London W12 0BZ, United Kingdom; MRC London Institute of Medical Sciences (LMS), London W12 0NN, United Kingdom; Institute of Clinical Sciences (ICS), Faculty of Medicine, Imperial College London, London W12 0NN, United Kingdom; Department of Mathematics, Imperial College London, London SW7 2BX, United Kingdom; Department of Mathematics, Imperial College London, London SW7 2BX, United Kingdom

## Abstract

**Motivation:**

Gene expression is characterized by stochastic bursts of transcription that occur at brief and random periods of promoter activity. The kinetics of gene expression burstiness differs across the genome and is dependent on the promoter sequence, among other factors. Single-cell RNA sequencing (scRNA-seq) has made it possible to quantify the cell-to-cell variability in transcription at a global genome-wide level. However, scRNA-seq data are prone to technical variability, including low and variable capture efficiency of transcripts from individual cells.

**Results:**

Here, we propose a novel mathematical theory for the observed variability in scRNA-seq data. Our method captures burst kinetics and variability in both the cell size and capture efficiency, which allows us to propose several likelihood-based and simulation-based methods for the inference of burst kinetics from scRNA-seq data. Using both synthetic and real data, we show that the simulation-based methods provide an accurate, robust and flexible tool for inferring burst kinetics from scRNA-seq data. In particular, in a supervised manner, a simulation-based inference method based on neural networks proves to be accurate and useful when applied to both allele and nonallele-specific scRNA-seq data.

**Availability and implementation:**

The code for Neural Network and Approximate Bayesian Computation inference is available at https://github.com/WT215/nnRNA and https://github.com/WT215/Julia_ABC, respectively.

## 1 Introduction

Gene expression is stochastic in nature due to the random timing of chemical reactions involving low numbers of key molecular players, such as genes and mRNAs, as well as the coupling to other variable cellular processes, such as the cell cycle. This stochasticity gives rise to cell-to-cell phenotypic variability in a population of genetically identical cells, with a broad impact on cellular functions.

Over the last 20 years, a considerable body of research combining experimental and mathematical studies has provided a deep understanding of the sources and consequences of this kind of biomolecular noise (Raj and Van Oudenaarden 2008, Shahrezaei and Swain 2008b, Sanchez and Golding 2013). Single-cell imaging studies of fluorescently tagged proteins were the first to quantify gene expression noise (Elowitz *et al.* 2002). Pioneering experimental and mathematical research broadly classified the sources of stochastic gene expression as either intrinsic due to random timing of the reactions involved in gene expression or as extrinsic due to the fluctuations of other relevant cellular factors (Swain *et al.* 2002). Also, direct time-lapse imaging and inference from snap-shot data revealed that gene expression could occur in bursts (Golding *et al.* 2005, Chubb *et al.* 2006, Raj *et al.* 2006, Suter *et al.* 2011, Stavreva *et al.* 2019). Methods such as the single-molecule Fluorescence In Situ Hybridization (smFISH) and the MS2 system allowed for the quantification of the gene expression noise and burstiness at the mRNA level (Vera *et al.* 2016, Bahrudeen *et al.* 2019). Most recently, the development of single-cell RNA sequencing (scRNA-seq) has made it possible to map global transcript counts in many cells and many genes routinely and cheaply (Eling *et al.* 2019). scRNA-seq data can reveal biophysical mechanisms of gene regulation when they are combined with mechanistic models (Gorin and Pachter 2020, Luo *et al.* 2023). However, due to additional technical variability in scRNA-seq data, inferring burst kinetics from such data is a challenging mathematical and statistical problem (Eling *et al.* 2019).

As mRNA copy numbers are typically low, it is generally well accepted that transcription is dominated by intrinsic noise (Raj *et al.* 2006), but the cell cycle can contribute to extrinsic expression noise (Thomas 2019). Recent work has shown that transcription is coupled to cell size in eukaryotic systems, which underlies mRNA concentration homeostasis and also underlies extrinsic variability in gene expression (Kempe *et al.* 2015, Padovan-Merhar *et al.* 2015, Ietswaart *et al.* 2017, Sun *et al.* 2020). Accounting for cell size and cellular context transcription is reported to be nonbursty following a Poisson distribution in some cellular systems (Battich *et al.* 2015, Sun *et al.* 2020). However, more generally transcription is observed to be bursty and is modelled well using a so-called telegraph model, in which transcription switches between on and off states (Raj *et al.* 2006). The telegraph model is theoretically extensively analysed, and it is known that it admits a Beta-Poisson distribution at steady-state (Peccoud and Ycart 1995, Kepler and Elston 2001, Raj *et al.* 2006, Shahrezaei and Swain 2008a, Kim and Marioni 2013). At the bursty limit of transcription, the solution of the telegraph model can be approximated as a negative-binomial distribution characterized by the burst size and burst frequency (Raj *et al.* 2006, Shahrezaei and Swain 2008a, Kumar *et al.* 2015, Amrhein *et al.* 2019, Thomas 2020). Moreover, the negative binomial (NB) distribution is a versatile over-dispersed distribution that is commonly used in bulk and scRNA-seq studies to model gene expression capturing both biological and technical dispersion (Anders and Huber 2012, Love *et al.* 2014, Tang *et al.* 2020, Svensson 2020).

The inference of parameters of mathematical models of stochastic gene expression from single-cell data is an important and challenging problem. Depending on the type of model, type of data, and the form of extrinsic noise, a range of different approaches have been developed recently to tackle this kind of inference problem (Lillacci and Khammash 2013, Neuert *et al.* 2013, Zechner *et al.* 2014, Fröhlich *et al.* 2016, Lenive *et al.* 2016, Schnoerr *et al.* 2017, Tiberi *et al.* 2018, Sun *et al.* 2020, Davidović *et al.* 2022, Fu *et al.* 2022). The inference of gene expression burst kinetics from scRNA-seq data has its own unique challenges due to specific kind of technical variability, complexity and sparsity of such data. Several recent studies have used single-allele-specific scRNAs-seq data to map global burst kinetics genome-wide based on the Beta-Poisson distribution solution of the telegraph model (Kim and Marioni 2013, Reinius *et al.* 2016, Jiang *et al.* 2017, Larsson *et al.* 2019). However, it is still an open question how to take into account the extrinsic biological and technical variability such as variation in cell size and capture efficiency in such methods (Blasi *et al.* 2017). The model by Jiang *et al.* (2017) considers the cell-specific variations via spike-ins data, which is an experimental control that is not commonly available. In addition, the model by Jiang *et al.* (2017) does not properly account for low and variable capture rates in scRNA-seq protocols. Meanwhile, the recent work by Larsson *et al.* (2019) applies Maximum Likelihood Estimation (MLE) directly on the raw scRNA-seq counts, hereby ignoring the cell-specific extrinsic variations. Ignoring extrinsic noise in such inference can inflate the amount of variability attributed to intrinsic noise and could lead to misleading estimates of the burst kinetics.

Here, we revisit the problem of statistical inference of the parameters of gene expression from scRNA-seq data focusing on the role of extrinsic variability. We present a mathematical model of gene expression measured by scRNA-seq. Our model appropriately accounts for the extrinsic variability introduced by cell-to-cell variations in scRNA-seq data through the capture efficiency and cell size. To estimate the gene-specific kinetic parameters, we implement and compare four different inference schemes: MLE, methods of moments estimation (MME), an Approximate Bayesian Computation (ABC) rejection sampling algorithm, and using direct likelihood-free inference based on a neural network (NN) implementation (Jørgensen *et al.* 2022). We benchmark these inference methods in a series of applications to synthetic and real data and discuss which methods work best.

## 2 Materials and Methods

### 2.1 Theory and model

**Figure 1.**
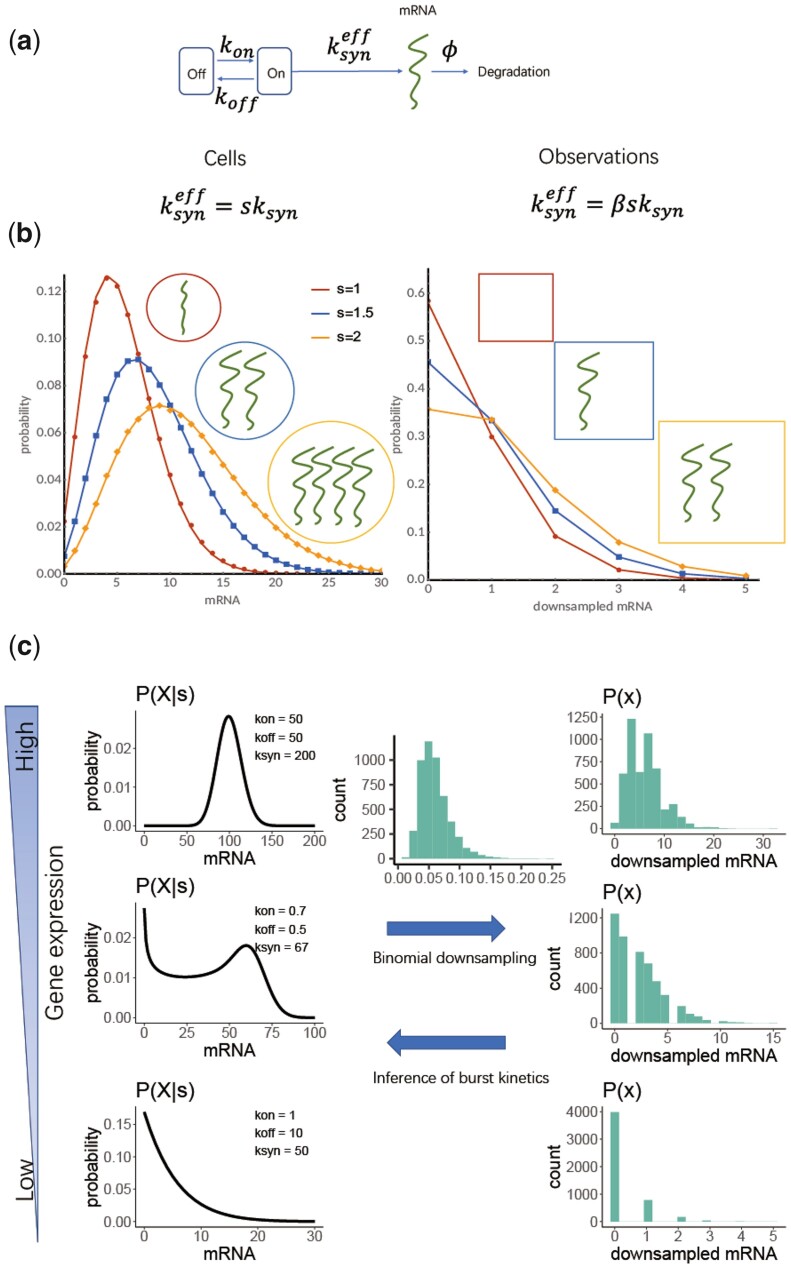
Model of stochastic gene expression and the effect of the cell size and sequencing capture efficiency on observed transcript count distributions. (a) An illustration of the telegraph model of stochastic gene expression and its associated parameters. The gene switches between an inactive and active state, and mRNAs are transcribed only from the active state. (b) Illustration of downsampling in scRNA-seq with a constant β=0.5. (Note that in reality, β tends to be smaller and varies across the cells.) The effective transcription rate ($k_{\text{syn}}^{\text{eff}}$) is proportional to the cell size in the original transcript counts (right) and both the cell size and capture efficiency in the observed counts (right). (c) Distributions of original mRNA counts in cells with constant size for three specific parameter sets for the telegraph model (left) and their corresponding downsampled distribution (right). The distribution of cell-specific capture efficiencies (β) used in downsampling is illustrated in the middle upper arrow (sampled from a log-normal distribution as described in Supplementary Section S1.3.3). The challenge lies in using the downsampled observed count distribution that is also affected by variability in the capture efficiency and cell size to infer the parameters of the original distribution (middle lower arrow).

The classical model for stochastic gene expression is the so-called telegraph model (Fig. 1a). It is known that the chemical master equation of the telegraph model results in a Beta-Poisson distribution for the mRNA at steady state (Peccoud and Ycart 1995, Raj *et al.* 2006, Iyer-Biswas *et al.* 2009).

However, the statement that the telegraph model results in a simple Beta-Poisson distribution is only valid in the absence of any extrinsic noise and cell cycle effects when considering a gene with a constant transcription rate ($k_{\text{syn}}$). These assumptions do not hold true for real-world applications. As discussed in the introduction, gene expression is coupled to cell size and is, therefore, affected by the cell cycle (Battich *et al.* 2015, Sun *et al.* 2020). Moreover, we have recently shown that the telegraph model satisfies the so-called stochastic concentration homeostasis condition when the transcription rate scales with cell size (*s*) (Thomas and Shahrezaei 2021). This notion implies that the transcript counts ($X_{ij}$) of gene *i* in cell *j* in a population of growing and dividing cells (Fig. 1) is distributed as follows: where $s_{j}$ is the cell size, and $k_{x,i}^{\prime }=k_{x,i}/(\phi_{i}+\alpha )$ denotes the gene-specific synthesis and promoter switching rates scaled by the effective degradation rate. The latter comprises the gene-specific degradation rate $\phi_{i}$ and the exponential growth rate α of the population.


(1) $$\begin{array}{l} X_{ij}∼\text{Poisson}(s_{j}k_{\text{syn},i}^{\prime }p_{i}),\\ p_{i}∼\text{Beta}(k_{\text{on},i}^{\prime },k_{\text{off},i}^{\prime }), \end{array}$$

During scRNA-seq, only a fraction of transcripts in each cell is captured. As we have recently demonstrated, the transcript counts observed in scRNA-seq data can be well-modelled by a binomial model with a cell-specific capture efficiency (probability) denoted by $\beta_{j}$ (Tang *et al.* 2020). Intuitively, the binomial model is a natural choice as each transcript in a given cell is captured with the same cell-specific probability $\beta_{j}$. Notably, the binomial model can explain the statistics of drop-out events without the need to invoke any zero-inflation models (Tang *et al.* 2020, Svensson 2020).

Using this binomial model, one can show that the distribution of observed transcripts ($x_{ij}$) in a cell of size $s_{j}$ and capture efficiency $\beta_{j}$ still follows the Beta-Poisson distribution but with a scaled effective synthesis rate: with $k_{\text{syn},i}^{\text{eff}}(\beta ,s)=\beta_{j}s_{j}k_{\text{syn},i}^{\prime }$ denoting an effective transcription rate for the observed counts. The observed counts *x* are necessarily lower than the actual original counts *X* and we therefore also refer to these as the downsampled counts. The dependence of the actual and observed transcript distributions on β and *s* is illustrated in Fig. 1b and c. This distribution then represents the correct likelihood function that should be used in the inference of kinetic rates from scRNA-seq data as it takes the biological variability introduced by the cell size and technical variability introduced by the capture efficiency into account. In the following, the kinetic rates of the model are defined relative to the effective decay rate, and as we are dealing with snap-shot data (and assuming a steady state), we will omit the primes on the scaled rates.


(2) $$\begin{array}{l} x_{ij}∼\text{Poisson}(k_{\text{syn},i}^{\text{eff}}(\beta_{j},s_{j})p_{i}),\\ p_{i}∼\text{Beta}(k_{\text{on},i}^{\prime },k_{\text{off},i}^{\prime }) \end{array}$$

Detailed descriptions of the likelihood-based and simulation-based inference methods used in our study can be found in Supplementary Section S1.

## 3 Results

### 3.1 Benchmarking on synthetic data

Our aim is to infer the parameters of the classic model of stochastic gene expression, the telegraph model (Fig. 1a), from scRNA-seq data. As illustrated in Fig. 1b, gene expression is coupled to the cell size, and scRNA-seq observations are affected by heterogeneous cell-specific capture efficiencies inherent to scRNA-seq protocols. This makes inference of the parameters of the gene expression, also referred to as burst kinetic parameters in this study, from downsampled scRNA-seq data a challenging task (as illustrated in Fig. 1c). As discussed in Section 2, the inference methods we are considering firstly include the existing bare maximum likelihood (BMLE) and bare method of moments estimation (BMME), in which raw scRNA-seq counts are used for inference (Larsson *et al.* 2019). In this study, we have introduced modified MLE and MME methods (denoted simply as MLE and MME), where the variability in the cell size and capture efficiency is taken into account in an approximate manner (see Supplementary Sections S1.2.1 and S1.2.2). We have also introduced two likelihood-free approaches, the approximate Bayesian computation rejection sampling scheme (ABC) and a direct inference approach based on Bayesian neural networks (NN) recently employed by Jørgensen *et al.* (2022) and based on Gal and Ghahramani (2016), Kendall and Gal (2017), and Kendall *et al.* (2017) (see Supplementary Sections S1.3.1 and S1.3.2). We note that in the ABC and NN methods, the cell size and capture efficiency has been taken into account by binomial down-sampling of the simulated gene expression using an effective capture deficiency (see Section 2 and Supplementary Sections S1.3.1 and S1.3.2).

We begin the result section by benchmarking the performance of the different inference methods on synthetic datasets that are generated from known gene-specific parameter sets as discussed in Supplementary Section S1.3.3. By comparing the inferred parameter sets to the ground truth, this section thus presents a self-consistency check that allows for an evaluation of the different methods in ideal settings. The synthetic datasets include different numbers of cells, spanning from 200 to 5000. In each case, we sample between 1000 and 7000 different combinations of kinetic parameters, repeating each combination 20 times.

**Figure 2.**
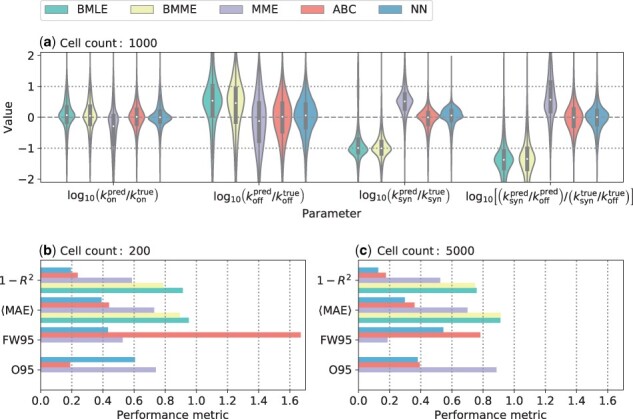
Comparison between different modelling approaches for allele-specific synthetic data with $\bar{\beta }=0.06$. (a) Logarithmic residuals across all four parameters for a dataset containing 1000 cells. (b, c) The panels contain four performance metrics for data containing 200 and 5000 cells, respectively. These metrics are the coefficient of determination ($R^{2}$), the mean absolute error (MAE), the fraction of the true parameter values that lie outside of the 95% confidence intervals (O95), and the width of the 95% confidence intervals in logarithmic space (FW95). Only the NN, ABC, and MME supply confidence intervals. For each number of cells, the synthetic dataset contains 7000 genes with 20 repetitions each. All metrics (except for FW95) are formulated such that a lower value implies a better fit. Note that the modified MLE is omitted from this summary as our implementation suffers from numerical issues (see Supplementary Fig. S5).

When assuming a fixed capture efficiency of 1.0, we find that all methods yield accurate and precise predictions for single-allele data. We summarize the results of this analysis in Supplementary Figs S2–S4 in the supplementary material. However, this scenario is not realistic; in real-world experiments, the capture efficiency is variable and much lower than one. So, next, we created another synthetic dataset for single-allele measurements with $\bar{\beta }=0.06$ (Klein *et al.* 2015, Tang *et al.* 2020). For this dataset, we find that the BMLE and BMME procedures by Larsson *et al.* (2019) lead to a pronounced systematic bias (offset) between the predictions and the ground truth for $k_{\text{off}}$ and $k_{\text{syn}}$ as well as the ratio of the two (Fig. 2). As a result, the scores of many performance metrics, including the mean squared and absolute errors, fall below those obtained from randomly assigning values to these parameters (Supplementary Figs S7 and S8). This makes sense as these methods effectively assume that the capture efficiency is 100% by not considering any normalization. Moreover, both the BMLE and BMME procedures fail to attribute parameter values to a large fraction (about 65%) of the dataset, yielding no parameter estimates and also producing outliers when the optimization methods fail. We note that while the modified MLE correctly includes capture efficiencies and therefore does not suffer from the systematic bias observed in BMLE, it suffers from numerical problems in the evaluation of the modified likelihood and the optimization (see Supplementary Figs S5 and S6). In contrast, our simulation-based approaches, rejection ABC and the NN, consistently yield accurate and precise predictions across all datasets and kinetic parameters. They thus consistently yield the lowest mean absolute and mean squared errors among all six methods, and the true values lie within the assigned 95% confidence interval of both methods in the majority of cases (Fig. 2).

As seen in Fig. 2, the accuracy of inferring $k_{\mathrm{off}}$ is the poorest among the kinetic parameters, suggesting some degree of nonidentifiability. Also, as expected, increasing the number of cells from 200 to 5000 improves the performance metrics of all methods. Interestingly, the NN has the best performance at small cell numbers. We also note that only the MME, ABC, and NN attribute confidence intervals while the remaining methods solely provide the best fit (Supplementary Figs S4 and S8). The MME generally leads to narrower confidence intervals than both the ABC and NN, but a significantly larger fraction of the true values do not lie within the error bars of the MME, suggesting that the MME significantly underestimates the prediction error.

Finally, we developed a modified MME, ABC, and NN method that works for nonallele-specific data (Supplementary Section S1.4) and benchmarked their performance on synthetic nonallele-specific data. We find that the NN yields smaller residuals than the other methods. The results are summarized in Supplementary Fig. S9 in the Supplementary Material. So, overall, we propose that the NN method is the most robust approach, and we mostly use this approach in the applications to real data in the rest of this study.

### 3.2 Sparsity of gene counts leads to wrong model identification

Even if expression counts are drawn from a Beta-Poisson distribution, the counts may equally well be fitted by other distributions depending on the underlying parameters. For example, the Beta-Poisson distribution reduces to a negative binomial distribution for large $k_{\text{on}}$; and to a Poisson distribution when the effective synthesis rate, and consequently the mean, is very small (Shahrezaei and Swain 2008a, Thomas 2020, Ham *et al.* 2021). These alternative distributions have fewer parameters than the Beta-Poisson distribution. We hypothesized that these identifiability issues could be exacerbated in scRNA-seq data through low capture efficiency (Fig. 1c).

To investigate how this aspect affects practical parameter identification, we use the Akaike information criterion (AIC), which is a commonly used metric for model selection and accounts for both the quality of the fit (the likelihood of the data) and the complexity of the model (the number of parameters). We generated a simulated dataset (500 cells and 7000 genes) using the Beta-Poisson model, and we calculated the AIC using the following three models and parameter choices for each gene:

- Beta-Poisson: Ground truth parameters were used.
- Negative binomial: R package bayNorm (Tang *et al.* 2020) (an NB model for nonallele specific scRNA-seq data) was applied to the raw counts to infer the NB parameters for calculating the AIC.
- Poisson: Raw counts were scaled by $\hat{\beta }$, after which the mean expression of each gene was calculated. For each gene in each cell, the mean expression was multiplied by $\hat{\beta }$ to be the mean parameter in the Poisson distribution, which was used for calculating the Poisson model AIC.

**Figure 3.**
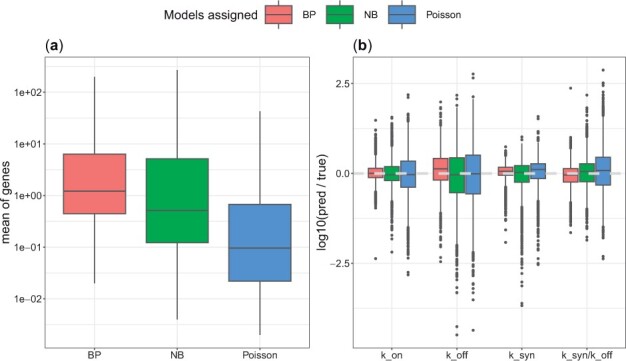
Genes with low counts are assigned to simpler models. (a) Based on synthetic data generated by the Beta-Poisson model, genes were labelled to be from one of the three models according to their AIC value. The mean counts for genes assigned to each model are shown. (b) The ratios between inferred and true parameter values in each group of genes are shown. Estimates from genes which are correctly assigned to the BP model are closer to ground truth values.

The genes were then assigned to the one among the three models (Beta-Poisson, negative binomial or Poisson) that yielded the lowest AIC value (Notably this model preference is strong as illustrated by the distribution of AIC weights, see Supplementary Fig. S10). As the data were generated from a Beta-Poisson model, one might expect this model to always be selected; however, for many genes, we found that one of the simpler models performed better based on the AIC score. This can be also visually inspected for a sample of genes, where simpler models have a likelihood very similar to data (see Supplementary Figs S11–S13). The genes for which the Poisson and NB models were preferred tend to have a lower mean expression (Fig. 3a), which highlights the fact that there is less information for estimating the Beta-Poisson parameters. Indeed, $k_{\text{syn}}$, which regulates the mean expression, has the highest impact on the identifiability of the Beta-Poisson model (Supplementary Fig. S14). This indicates that inference of burst kinetics is only possible for genes that have a sufficiently high expression—which was to be expected. In line with this result, we observe that the inference accuracy is poorer for the lowly expressed genes in our synthetic data (Fig. 3b, Supplementary Fig. S16). The same qualitative conclusions can be drawn via the Widely Applicable Information Criterion (WAIC) (Watanabe 2010) for model selection (Supplementary Fig. S15, and see Supplementary Section S1.4.1 for detailed formula for calculating WAIC via ABC), however, we observe in this case simpler models are less commonly selected. As WAIC results is expected to converge to AIC for large sample size and also it relies on ABC samples, we believe the discrepancy maybe due to sample size, or the effect of specific choice of prior or the approximate nature of ABC.

### 3.3 Application to real-world data

#### 3.3.1 Estimating kinetic parameters from individual allele data

We used the NN method to reassess the allele-specific data from Larsson *et al.* (2019) containing 10 727 genes and 224 cells. The data contain missing values. The number of missing values varies between different genes. Here, we only include genes with mean expression across nonmissing values above 1. As shown above, this is important as genes with low counts do not contain enough information. This first filtering leaves us with 1992 genes. Of these genes, we remove genes with a large number of missing values. This leaves us with 1953 genes. We find that the NN yields kinetic parameter estimates that are consistent with those obtained from the BMLE procedure by Larsson *et al.* (2019) when assuming that $\bar{\beta }=1.0$. However, as seen in Supplementary Fig. S17 using realistically small and cell-specific capture efficiencies leads to a systematic shift to higher burst sizes and a wider spread in burst frequency. The choice of prior used in training our NN inference method has only a small effect on the inference results, which suggests the robustness of our method (Supplementary Fig. S17). Also, we find as expected the distribution of cell specific $k_{\text{syn}}^{\text{eff}}$ to be wider and shifted to the lower compared to the distribution of $k^{\text{syn}}$ due to the relatively wide distribution of capture efficiencies (Supplementary Fig. S18).

**Figure 4.**
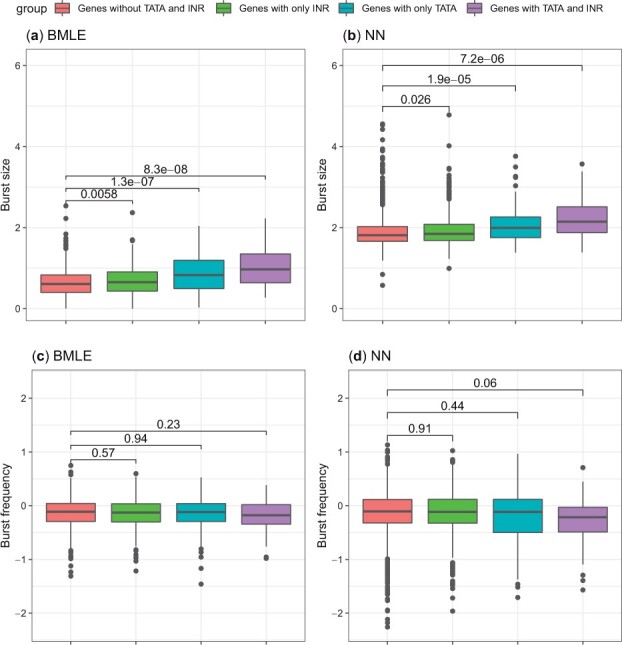
The relationship between burst kinetics and promotor characteristics based on the NN results with $\bar{\beta }=0.06$ and the BMLE from Larsson *et al.* (2019). Results are shown for Allele c57. (a, b) Box plots of burst size and (c, d) burst frequency estimates of genes with or without TATA elements and Inr. The *P*-values of the Wilcox test between groups are shown.

As investigated in the original study by Larsson *et al.* (2019), we look at the link between the presence of TATA elements and Initiator (Inr) and the burst kinetics using our inferred parameters. We find that the NN yields kinetic parameter estimates that are qualitatively consistent with those obtained from the original MLE procedure by Larsson *et al.* (2019) such that genes with TATA elements have larger burst size (Fig. 4a and b). By filtering out lowly expressed genes, our analysis reveals that genes with only Inr can boost burst sizes (Fig. 4). Similar qualitative results can be achieved via the MLE approach adapted by Larsson *et al.* (2019) after removing the lowly expressed genes (Fig. 4). We note that we do not observe consistent and significant results for the link between the presence of TATA elements and Inr and burst frequency using different inference methods (Fig. 4 and Supplementary Fig. S19).

Based on the present dataset, we find the simulations to successfully recover the observed relation between the dropout rate and the mean expression for each allele (Supplementary Fig. S20), providing further support for the accuracy of our mathematical model of scRNA-seq data.

Finally, in our inference methods, the only source of biological and technical variability is the cell size and capture efficiency. To test the validity of this assumption in real data, we simulated data for two independent alleles from 100 cells down-sampled by the same capture efficiency using parameters inferred by the NN method on the single-allele data of Larsson *et al.* (2019). We then computed the correlations between the two alleles in simulated data and plotted the results against the correlation between the two alleles in real data (Supplementary Fig. S21). We find a clear linear relationship between the simulated correlation and the real correlation. This indicates that it is reasonable to consider cell size and capture efficiency as an important source of extrinsic noise since we observe most genes to have a significantly positive correlation between the two alleles, captured in our simulations. These results suggest that the observed positive correlation between the gene expression between the two alleles is well-explained by variation in capture efficiency across cells. So, one does not need to invoke correlated activity between the alleles or other significant sources of extrinsic noise. This further motivates the approach we have proposed for the inference of gene expression parameters from nonallele-specific data. Interestingly, for some genes in the real data, there is a negative correlation between two alleles, which might indicate anti-correlation in the activity of those genes.

#### 3.3.2 Estimating kinetic parameters from nonallele-specific scRNA-seq data

In this section, we analyse scRNA-seq data of mouse brain cells from two recent studies (Mizrak *et al.* 2019, Ximerakis *et al.* 2019) to highlight the application of our inference methods (MME, ABC, and NN) on nonallele-specific data that assume that the counts are related to the sum of two identical but independent alleles (Supplementary Section S1.4).

The data from Mizrak *et al.* (2019) contain 28 407 cells from mouse brains (after removing doublets) and covers multiple cell types like neuronal progenitors [active neural stem cells, transit amplifying cells, and neuroblasts (aNSC+TAC+NB)], oligodendrocyte progenitor cells (OPCs), committed oligodendrocyte precursors (COPs), oligodendrocytes (OLG), microglia (MG), astrocytes (ASC), and neurons. In addition, we explored the data from mouse brains Ximerakis *et al.* (2019), where there are 37 069 cells collected from either young or old mice. The dataset contains various cell types, including neural stem cells (NSC), mature neurons (mNEUR), OPC, and other cell types from young and old mice.

**Figure 5.**
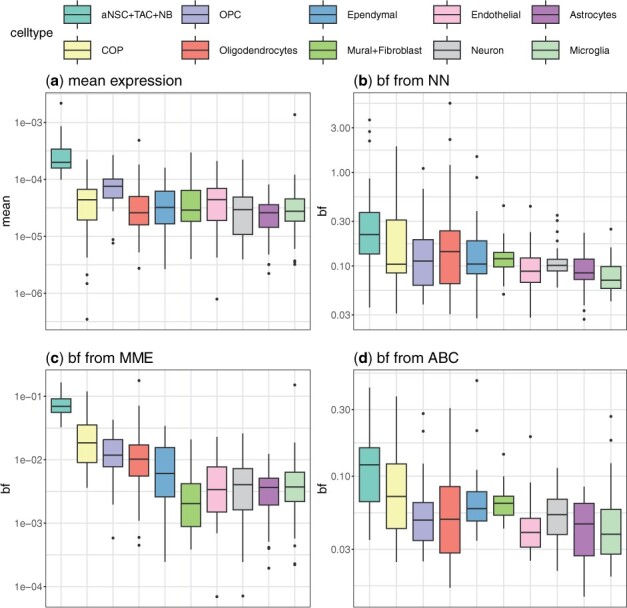
Higher expression of stem cell marker genes in stem cells is associated with higher burst frequency. Cell types are allocated using aNSC marker genes reported in Mizrak *et al.* (2019). The first three cell types are stem cell-like, and the rest are mature cell types. (a) Box plots of mean expression of stem cell markers across the cell types are shown. Mean expressions were calculated based on total count normalized data; Box plots of inferred burst frequencies using NN (b), MME (c), and ABC (d) inference approach.

Cell type markers are by definition the ones that are overexpressed in a particular cell type but not in others. Here, we investigate whether these gene expression alterations are associated with changes in burst size or burst frequency. When comparing stem cells (aNSC+TAC+NB) with other differentiated cells, all inference methods reveal higher burst frequencies for stem cell markers in stem cells than differentiated cells like neurons and oligodendrocytes (Fig. 5). A different visualization of these data in Supplementary Fig. S23 shows a clear correlation between the mean expression and burst frequency. To a lower degree, we see that the burst size increases with the mean expression (Supplementary Fig. S22) (though the burst size from MME is not consistent with that obtained from the ABC and NN). Interestingly, cells at different stages of oligodendrocyte differentiation (COP, OPC, and oligodendrocyte) tend to have either slightly higher or similar burst frequency/size to the other mature cell types.

**Figure 6.**
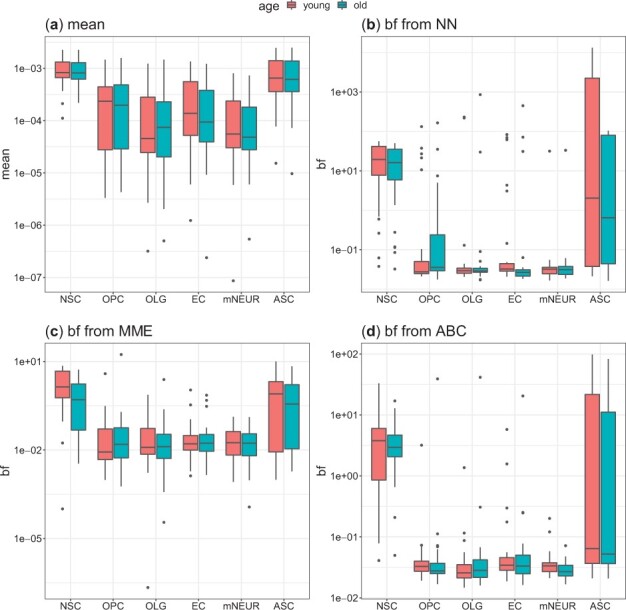
(a) Mean expression, calculated after the total count was normalized; Burst frequency estimated using NN (b), MME (c), and ABC (d). Here, we use the NSC marker genes reported in Ximerakis *et al.* (2019).

Our second dataset from Ximerakis *et al.* (2019) contains data from both young and old brains and supports the mentioned relationship between the mean expression and burst frequency (but not burst size) for the stem cell markers in stem cells regardless of brain age (Fig. 6 and Supplementary Fig. S24). Ximerakis *et al.* (2019) also reported that genes encoding ribosomal subunits have a reduced expression upon ageing. Here, we again ask whether it is the burst frequency or burst size this time in the ribosomal genes that changes following changes in the mean expression upon ageing in the different cell types. We find that, while changes in the mean expression of ribosomal genes in the young and old cells follow different trends in the stem/progenitor cells [NSC (Ximerakis *et al.* 2019), ASC (Clarke *et al.* 2018, Ximerakis *et al.* 2019), and OPC (Ximerakis *et al.* 2019)] compared with other mature cell types, results from NN, MME, and ABC show that as before mainly the burst frequency but not burst size is modified to regulate mean expression (Supplementary Figs S25 and S26).

## 4 Discussion

In this article, we revisited the problem of inferring the burst kinetics of gene expression from scRNA-seq data. We provide a novel expression for the likelihood to be used for single-allele scRNA-seq data, which allows us to take cell-to-cell variation in cell size and capture efficiency correctly into account. We show that numerical challenges can make maximum likelihood estimation (MLE) unreliable. To overcome this limitation, we introduce likelihood-free approaches, including a modified method of moments (MME) and two simulation-based inference methods. We demonstrate the reliability and flexibility of the simulation-based inference methods through a series of benchmarks on synthetic and real data. We show that these methods also provide confidence intervals and could be easily generalized to nonsingle-allele situations, which makes them more widely applicable. We obtain the best results using simulation-based inference based on Bayesian neural networks (Gal and Ghahramani 2016, Jørgensen *et al.* 2022). Our analysis suggests the importance of properly taking into account cell size and capture efficiency variation and can be used to guide the design of scRNA-seq experiments suitable for reliable estimates of gene expression parameters. While, as expected, more cells and more sequencing depth will yield better results, we find that about 1000–2000 cells are sufficient to estimate the burst kinetics accurately.

Recent studies have used the maximum likelihood estimation method (Larsson *et al.* 2019) or Bayesian method (Kim and Marioni 2013) using a Beta-Poisson model without any normalization. As we show in this article, this approach can result in biased and distorted distributions of estimates for burst kinetic parameters, including the burst size. Also, we show that burst kinetics parameters become unidentifiable for lowly expressed genes and that this property could result in misleading results. While maximum likelihood estimation has good theoretical guarantees, computational challenges in evaluating the likelihood and also challenges in optimization can make this method less favourable. Indeed, recent studies have likewise highlighted the challenges with maximum likelihood estimation and the nonidentifiability for similar models of stochastic gene expression (Ham *et al.* 2021, Fu *et al.* 2022).

There are few available allele-specific scRNA-seq datasets, but UMI-based nonallele-specific scRNA-seq data are highly abundant. We have therefore modified the MME method and our simulation-based methods to infer the kinetic parameters directly from nonallele-specific (e.g. UMI) count matrices. Although we assume that the two gene copies have identical kinetic parameters and transcribe independently in this study, we note that these assumptions can easily be relaxed for simulation-based methods. Indeed, some recent studies have suggested evidence for allelic imbalance and dependence in burst kinetics across the gene alleles in existing scRNA-seq data (Choi *et al.* 2019, Mu *et al.* 2021). We applied our methods to two mouse brain scRNA-seq datasets. Our results indicate that gene regulation across stem cells and the ageing of the brain tends to be associated with the regulation of burst frequency and, to some degree, burst size. A recent study has proposed that epigenetic regulation of burst frequency in fitness genes upon stress could underlie the evolution of cancer (Loukas *et al.* 2023).

We note here that we are neglecting other possible sources of extrinsic variability, such as fluctuations in the kinetic rates due to fluctuations of other molecules in the cells (Ham *et al.* 2021). However, we have shown here that many gene expression correlations between alleles can be explained by accounting for variations in cell size and capture efficiency. In fission yeast, we have previously shown that it is possible to capture most of the extrinsic variability observed in gene expression by accounting for cell size variation (Sun *et al.* 2020). Other studies have included the effect of different cell cycle stages, replication and gene copy numbers (Fu *et al.* 2022). Sun and Zhang (2020) used allele-specific expressions in diploid cells and intrinsic and extrinsic noise decomposition to study the genetic factors affecting gene expression noise. We note that more detailed mechanistic models of RNA-sequencing protocols can help to explain more of the technical noise and biases in the data (Dyer *et al.* 2019, Fischer *et al.* 2019, Davies *et al.* 2021, Gorin and Pachter 2022, Luo *et al.* 2023).

Inferring kinetic parameters of stochastic gene expression from scRNA-seq data is challenging. First and foremost, the data are sparse and have missing values. This characteristic of the data presents an obstacle to any attempt to estimate the parameters accurately. In addition, the extrinsic variables, such as cell size and capture efficiency, are usually not known [for an exception, where cell size has been measured along with scRNA-seq, see Saint *et al.* (2019)]. Furthermore, measurements or theoretical considerations that constrain the kinetic parameters’ range are not readily available. Statistical analysis, such as the one presented in this article, would thus benefit from additional measurements or other constraints that would provide tighter priors. While many researchers have already studied the inference of kinetic parameters from high-throughput data, such as scRNA-seq data, several aspects are hence, by far, not fully explored. An important area of future research is using multi-omic single-cell data. The data are quickly becoming available and could thus inform our understanding of global gene expression variability (Lee *et al.* 2020, Argelaguet *et al.* 2021). Some research is already starting in this important area based on both statistical data integration (Argelaguet *et al.* 2021, Rautenstrauch *et al.* 2022, Rodosthenous *et al.* 2021) and model-based inference (La Manno *et al.* 2018, Bergen *et al.* 2020, Gorin and Pachter 2022). Ultimately, by harnessing gene-gene correlations, such multi-omic single-cell datasets could be used to infer genetic networks (Stumpf 2021, Qiu *et al.* 2022).

In summary, we proposed a simple and accurate method to take the variation of cell size and capture efficiency into account when performing the inference of burst kinetics from scRNA-seq data. We provide implementations of our likelihood-free approaches that are robust and flexible and apply them to synthetic and real data. Our analysis shows how state-of-the-art inference tools can help us to extract valuable information missed by standard approaches.

## Supplementary Material

btad395_Supplementary_Data

## Data Availability

Regard to real experimental data used in this study, scRNA-seq allele specific data can be downloaded from https://github.com/sandberg-lab/txburst; scRNA-seq data from Mizrak study can be downloaded from GEO: GSE109447; scRNA-seq data from Ximerakis study can be downloaded from GEO: GSE129788.
