# Supplementary material for: Modelling capture efficiency of single-cell RNA-sequencing data improves inference of transcriptome-wide burst kinetics
Source: Bioinformatics. 2023 Jun 24;39(7):btad395. doi: 10.1093/bioinformatics/btad395 (PMC10318389; doi:10.1093/bioinformatics/btad395)
Supplement: btad395_Supplementary_Data [file btad395_supplementary_data.pdf]

S1 Supplementary methods

S1.1 Estimation of the capture efficiency

Let x_{ij} ($i \in \{1, 2, \ldots, P\}$ and $j \in \{1, 2, \ldots, Q\}$) denote the number of transcripts reported for the ith gene in the jth cell in a scRNA-seq study. Also let s_j and β_j denote the cell size and capture efficiency of cell j. The measurements could be single-allele or non-single allele, depending on the protocol. We introduce $\hat{\beta}_j = s_j \beta_j$ for cell j, which can simply be estimated using an appropriate cell-specific scaling factor [3] from the raw data (x_{ij}). One simple such measure of the cell-specific scale factor is the total number of counts:

$$T_j = \sum_i x_{ij}.$$

We posit that $\hat{\beta}_j = \frac{T_j}{\bar{T}} \bar{\beta}$ where $\bar{\beta}$ is the average capture efficiency in the scRNA-seq protocol, and $\bar{T}$ is the average total count across cells. It can be estimated, for example, from smFISH data [17, 14]. Throughout most of this paper, $\bar{\beta}$ is set to 0.06 in accordance with Klein *et al.* [10].

S1.2 Likelihood- and moment-based estimation of burst kinetics

S1.2.1 Maximum Likelihood Estimation (MLE)

Let dBP denote the Beta-Poisson distribution. A parameter estimation method proposed in the literature [11] is obtained by maximising the log-likelihood:

$$\ell_{ori,i} = \sum_{j=1}^{Q} \log(\text{dBP}(x_{ij}, k_{\text{on},i}, k_{\text{off},i}, k_{\text{syn},i})),$$

where x_{ij} are the raw single-allele scRNA-seq counts for gene i in cell j as above, and the sum runs over all cells in the data set — here, we assume that the observations are independent. This approach attributes all variations in the transcript counts to the intrinsic stochastic properties of gene expression (in this case, in the framework of the telegraph model). As this method does not use any normalisation and relies on raw scRNA-seq counts, we have termed it in this study as bare MLE (denoted as BMLE).

There are two ways to evaluate ℓ_{ori} numerically. First, one might employ the so-called integral method used in previous studies (see [7] and [11] for more details). Alternatively, one can use the analytic form derived by Raj *et al.* [13] and Amrhein *et al.* [1]:

$$\begin{aligned} &\text{dBP}(x_{ij} = n, k_{\text{on},i}, k_{\text{off},i}, k_{\text{syn},i}) \\ &= \frac{\Gamma(k_{\text{on,i}} + k_{\text{off,i}}) {k_{\text{syn,i}}}^n \Gamma(k_{\text{on,i}} + n)}{\Gamma(k_{\text{on,i}})\Gamma(n+1)\Gamma(k_{\text{on,i}} + k_{\text{off,i}} + n)} {}_1F_1(k_{\text{on,i}} + n, k_{\text{on,i}} + k_{\text{off,i}} + n, -k_{\text{syn,i}}). \end{aligned} \quad (1)$$

The limitation of this method is that it implicitly assumes perfect capture efficiency. This issue could in principle be overcome by considering the log-likelihood function derived from the model in (2) in the main text:

$$\ell_i = \sum_{j=1}^{Q} \log(\text{dBP}(x_{ij}, k_{\text{on},i}, k_{\text{off},i}, k_{\text{syn},i}\hat{\beta}_j)). \quad (2)$$

This method is denoted simply as MLE in this study (in contrast to BMLE). The integral method is not suitable for our modified MLE method, however, as we consider cell-specific parameters which would require us to marginalise over all cells. Since it is not possible to efficiently vectorize this approach, the computation becomes very time-consuming. We hence utilised the analytic form (1) to take cell-specific parameters into consideration. However, this method is numerically challenging since it involves the evaluation of Eq. (1) for various expression levels and parameter combinations. This circumstance can lead to uncontrolled numerical errors that impair the accuracy of the MLE (Fig. S1).

S1.2.2 Method of Moments Estimation (MME)

As an alternative to the MLE method, we consider the Method of Moments Estimation as presented by Larsson *et al.* [11]. In this study, we denote this approach as the bare MME (BMME) as it is based on raw counts. The method considers the first three moments M_1^i, M_2^i, and M_3^i of each gene. These moments can be estimated based on the raw single-allele scRNA-seq data (cf. [11]):

$$\begin{aligned} M_1^i &= \frac{1}{Q}\sum_{j=1}^{Q} x_{ij}, \\ M_2^i &= \frac{1}{Q}\sum_{j=1}^{Q} x_{ij}(x_{ij}-1), \\ M_3^i &= \frac{1}{Q}\sum_{j=1}^{Q} x_{ij}(x_{ij}-1)(x_{ij}-2). \end{aligned} \tag{3}$$

For brevity of notation, we will omit the index i from the moments as the parameters of each gene can be estimated independently. One can then define the quantities

$$\begin{aligned} r_1 &= M_1, \\ r_2 &= M_2/M_1, \\ r_3 &= M_3/M_2; \end{aligned} \tag{4}$$

that are related to the kinetic parameters:

$$\begin{aligned} k_{\text{on}} &= \frac{2r_1(r_3-r_2)}{r_1r_2-2r_1r_3+r_2r_3}, \\ k_{\text{off}} &= \frac{2(r_3-r_2)(r_1-r_3)(r_2-r_1)}{(r_1r_2-2r_1r_3+r_2r_3)(r_1-2r_2+r_3)}, \\ k_{\text{syn}} &= \frac{2r_1r_3-r_1r_2-r_2r_3}{r_1-2r_2+r_3}. \end{aligned} \tag{5}$$

To take variations in the capture efficiency ($\hat{\beta}$) into consideration, we propose a heuristic approach that uses $x_{ij}/\hat{\beta}_j$ instead of the raw x_{ij} in the estimation of the moments. We denote this method simply as MME in contrast to BMME.

S1.3 Simulation-based estimation of burst kinetics

S1.3.1 Approximate Bayesian Computation (ABC)

When dealing with our modified MLE method, there are numerical instability issues, and the optimisation is challenging. Meanwhile, it is known that MME may lead to biased estimates. Alternative methods that avoid these issues are likelihood-free approaches that imply sampling simulations. In our case, we sample from our analytical distribution (Eq. (2)).

Here, we employ ABC rejection sampling using priors that are constructed based on Section S1.3.3. In ABC, one relies on a distance measure between the data and the simulations. For this purpose, we employ the Hellinger distance since it has good properties with respect to model misspecification [4].

In the data, each gene's expression ($\hat{x}$) is a vector of scRNA-seq counts of length Q cells associated with another vector of capture efficiencies $\hat{\beta}$. For each gene, the algorithm goes through the following steps:

1. We draw kinetic parameter sets from the prior: $\boldsymbol{\theta}^\star \sim \pi(\boldsymbol{\theta})$ (Section S1.3.3). Some parameter sets are filtered out to make sure that $M_1 \in \{M_{5\%}, M_{95\%}\}$, where $M_{5\%}$ and $M_{95\%}$ indicate the 5th and 95th percentiles of 1,000 M_1 estimates from bootstrapped MME (that is, we sample cells with replacement 1,000 times and hence obtain 1,000 M_1 estimates for each gene).

2. We simulate data ($x^\star$) by sampling a vector of gene expressions from Eq. (2) in the main text using $\boldsymbol{\theta}^\star$ and a cell-specific vector for $\hat{\beta}$.

3. We calculate the Hellinger distance between the data ($\hat{x}$) and the model predictions ($x^\star$: $H = d(x, x^\star)$).

4. We repeat the steps above $10,000$ times, whereby we obtain a vector of distances, $\boldsymbol{H} = (H_1, H_2, \ldots, H_{10,000})$. The parameter sets within the lowest $5\,\%$ of distances are accepted.

5. Lastly, we use the medians of the accepted parameter sets as point estimates for the three kinetic parameters.

S1.3.2 Direct likelihood-free inference based on neural networks (NN)

Recently, machine learning approaches are finding applications in likelihood-free inference [2]. In one such approach that we have recently adopted [8] (here denoted by NN), we train a Bayesian neural network using sets of parameters sampled from data and their corresponding summary statistics of the simulated output. We employ a broad deep neural network with 3 hidden layers with 100 neurons each. To avoid overfitting, we invoke early stopping and dropout during the training process [6]. By also including dropout during the inference phase in tandem with the loss function presented by Gal and Ghahramani [5], approximating a Bayesian neural network, we can furthermore access the uncertainty associated with the parameter predictions [see also 9]. So, given summary statistics of the data, be it synthetic or real data, the trained neural network produces samples from the approximate posterior distribution.

As in the case of the rejection ABC, we simulate data by sampling gene expression counts based on the desired kinetic parameter values and subsequently downsampling the counts based on the capture efficiency. The training and test data for the NN are thus constructed analogously to the synthetic data used in the self-consistent tests in Section 3.1. For data with 5000 cells, our NN is

trained on 10,000 simulations drawn from the uniform Fano prior discussed in Section S1.3.3. 4000 additional samples were drawn for the validation of the network. We note that the performance of the NN is only marginally altered when reducing the training set to 1000 simulations. When dealing with data with lower cell counts, we increase the number of simulated genes in the training and validation sets proportionally to the relative decrease in the cell count. When dealing with data with, say, 500 cells as opposed to 5000 cells, the number of genes in the training set is hence increased from 10,000 to 100,000, while 40,000 rather than 4000 genes are used in the validation set.

The NN draws on 15 summary statistics. Three of these measures relate to the raw reported gene expression after downsampling. These measures are the logarithm of the mean number of reported transcripts, the logarithm of the sum of reported transcripts, and the fraction of zeros in the scRNA-seq data. For the remaining twelve summary statistics, we estimate the capture efficiency based on the data and scale the reported gene expression based on this estimate. The summary statistics that rely on the scaled gene expression include the logarithm of the range of scaled transcript count, the logarithm of the highest scaled transcript count, the 10th percentile, the 25th percentile, the median transcript count, the 75th percentile, the 95th percentile, the logarithm of the variance, the skewness (3rd moment), the kurtosis (4th moment), the logarithm of the coefficient of variation, and the logarithm of the total number of scaled transcript counts. All logarithms are base 10. With this wide range of summary statistics, we aim to capture most of the information present in the scRNA-seq data. The compression of distributions into a set of key features thus often comes with the loss of relevant information (cf. a discussion on the sufficiency of summary statistics by [16]). However, we also note the performance is not dramatically compromised by dropping some of these summary statistics.

S1.3.3 Choice of priors in simulation-based methods

We need a choice of prior for the parameters of the model for the ABC method. The same priors are used to train the NN method and also to generate synthetic data for benchmarking. We have chosen these priors in a specific way to create reasonable parameter sets. In this paper, these kinetic parameters that enter Eq. (2) in the main text are computed in the following steps. First, we draw the logarithm of k_{on} from a uniform prior:

$$k_{\text{on}} \sim 10^{\mathcal{U}(\log_{10}(0.01),\, \log_{10}(100))} \tag{6}$$

Secondly, we draw the ratio between k_{on} and k_{off} from a normal prior with a mean of 0.05 and a standard deviation of 0.5:

$$k_{\text{off}} = \frac{k_{\text{on}}}{r}, \quad r \sim |\text{Normal}(\mu = 0.05, \sigma = 0.5)|. \tag{7}$$

Finally, we compute k_{syn} based on the values of k_{on} and k_{off} as well as the Fano factor (F; variance over mean, for constraining parameter sets to lie in a reasonable range.):

$$k_{\text{syn}} = \frac{(F-1)(k_{\text{on}} + k_{\text{off}})(k_{\text{on}} + k_{\text{off}} + 1)}{k_{\text{off}}}. \tag{8}$$

Here, we assume that the prior on the logarithm of the Fano factor is uniform:

$$F \sim 10^{\mathcal{U}(\log_{10}(1.001),\, \log_{10}(30))}.$$

Given a parameter set $\boldsymbol{\theta} = (k_{\text{on}}, k_{\text{off}}, k_{\text{syn}})$, we simulate the outcome for a predetermined number of cells by drawing from a Beta-Poisson distribution. We then downsample the synthetic data based on the capture efficiency, β. When simulating synthetic data, we either employ a fixed value for all cells, mirroring the approach by Larsson *et al.* [11], or draw individual values for β for each cell from a log-normal distribution. For this purpose, we draw samples from a log-normal distribution with a mean of 2.74 and a standard deviation of 0.39 [17]. This distribution is subsequently scaled to have a mean of $\bar{\beta}$.

S1.4 Extension to non-allele-specific counts

The previous methods can be applied to allele-specific scRNA-seq data since they rely on a model for single genes based on the telegraph model. To go beyond this limitation, we modified the methods discussed above so that they can be directly applied to non-allele-specific scRNA-seq data that measures the sum of transcript counts from both alleles for each gene. To this end, we have made the simplifying assumption that the two gene alleles share the same parameters and their expression is independent of one another.

For MLE applied to non-allele-specific data, including protocols based on Unique molecular identifiers (UMI)[15], the likelihood is the distribution of observed counts of two independent integer-valued random variables (X=Y+Z), which can be obtained as follow: $\text{dBP}_{\text{UMI}}(X = x) = \sum_{k=0}^{x} \text{dBP}(Y = k)\text{dBP}(Z = x - k)$. It follows that $\ell = \sum \log(\text{dBP}_{\text{UMI}}(x))$ for optimization, where the summation is taken across cells. However, for our purposes, it is not efficient to evaluate the log-likelihood in this manner. We hence omit MLE when dealing with non-allele-specific data.

In order to apply MME to non-allele-specific counts, the procedure can be modified by replacing M_1, M_2 and M_3 in Eq. (4) with m_1, m_2 and m_3 defined below:

$$\begin{aligned} m_1 &= M_1/2, \\ m_2 &= M_2/2 - 2m_1, \\ m_3 &= M_3/2 - 3m_1 m_2. \end{aligned} \tag{9}$$

With regards to rejection ABC and the training data for the NN, we simulate non-allele-specific data by taking the sum of two random variables drawn independently from a Beta-Poisson distribution and subsequently downsampling the total count.

S1.4.1 Calculation of Widely Applicable Information Criterion (WAIC)

Suppose for each gene, there are S parameter sets ($\boldsymbol{\theta} = (k_{\text{on}}, k_{\text{off}}, k_{\text{syn}})$) drawn from a posterior distribution (both NN and ABC allows for generating parameter sets from posterior) based on Q cells. We have

$$\begin{aligned} \text{WAIC} &= T_Q + \frac{V_Q}{Q}, \text{where} \\ T_Q &= -\frac{1}{QS}\sum_{i=1}^{Q}\sum_{j=1}^{S} \log P(x_i|\boldsymbol{\theta}_j), \text{and} \\ V_Q &= \sum_{i=1}^{Q}\{\frac{1}{S}\sum_{j=1}^{S}(\log P(x_i|\boldsymbol{\theta}_j))^2 - (\frac{1}{S}\sum_{j=1}^{S}\log P(x_i|\boldsymbol{\theta}_j))^2\}. \end{aligned} \tag{10}$$

P denotes either BP, NB or Poisson distribution. Note that given BP parameter sets $\boldsymbol{\theta} = (k_{\text{on}}, k_{\text{off}}, k_{\text{syn}})$, we can convert it to NB or Poisson parameters:

- NB: $\mu = \frac{k_{\text{syn}} k_{\text{on}}}{k_{\text{on}} + k_{\text{off}}}$ and size k_{on};
- Poisson: $\mu = \frac{k_{\text{syn}} k_{\text{on}}}{k_{\text{on}} + k_{\text{off}}}$.

References

[1] Amrhein, L., Harsha, K., and Fuchs, C. (2019). A mechanistic model for the negative binomial distribution of single-cell mrna counts. *bioRxiv*.

[2] Cranmer, K., Brehmer, J., and Louppe, G. (2020). The frontier of simulation-based inference. *Proceedings of the National Academy of Sciences*, **117**(48), 30055–30062.

[3] Eling, N., Morgan, M. D., and Marioni, J. C. (2019). Challenges in measuring and understanding biological noise. *Nature Reviews Genetics*, **20**(9), 536–548.

[4] Frazier, D. T. (2020). Robust and efficient approximate bayesian computation: A minimum distance approach. *arXiv preprint arXiv:2006.14126*.

[5] Gal, Y. and Ghahramani, Z. (2016). Dropout as a bayesian approximation: Representing model uncertainty in deep learning. In *International conference on machine learning*, pages 1050–1059. PMLR.

[6] Hinton, G., Krizhevsky, A., Sutskever, I., and Salakhutdinov, R. (2014). Dropout: A simple way to prevent neural networks from overfitting. *Journal of Machine Learning Research*, **15**, 1929–1958.

[7] Jiang, Y., Zhang, N. R., and Li, M. (2017). Scale: modeling allele-specific gene expression by single-cell rna sequencing. *Genome biology*, **18**(1), 74.

[8] Jørgensen, A. C. S., Ghosh, A., Sturrock, M., and Shahrezaei, V. (2022). Efficient bayesian inference for stochastic agent-based models. *PLOS Computational Biology*, **18**(10), e1009508.

[9] Jospin, L. V., Buntine, W., Boussaid, F., Laga, H., and Bennamoun, M. (2020). Hands-on bayesian neural networks–a tutorial for deep learning users. *arXiv preprint arXiv:2007.06823*.

[10] Klein, A. M., Mazutis, L., Akartuna, I., Tallapragada, N., Veres, A., Li, V., Peshkin, L., Weitz, D. A., and Kirschner, M. W. (2015). Droplet barcoding for single-cell transcriptomics applied to embryonic stem cells. *Cell*, **161**(5), 1187–1201.

[11] Larsson, A. J., Johnsson, P., Hagemann-Jensen, M., Hartmanis, L., Faridani, O. R., Reinius, B., Segerstolpe, Å., Rivera, C. M., Ren, B., and Sandberg, R. (2019). Genomic encoding of transcriptional burst kinetics. *Nature*, **565**(7738), 251.

[12] Mizrak, D., Levitin, H. M., Delgado, A. C., Crotet, V., Yuan, J., Chaker, Z., Silva-Vargas, V., Sims, P. A., and Doetsch, F. (2019). Single-cell analysis of regional differences in adult v-svz neural stem cell lineages. *Cell reports*, **26**(2), 394–406.

[13] Raj, A., Peskin, C. S., Tranchina, D., Vargas, D. Y., and Tyagi, S. (2006). Stochastic mrna synthesis in mammalian cells. *PLoS biology*, **4**(10).

[14] Saint, M., Bertaux, F., Tang, W., Sun, X.-M., Game, L., Köferle, A., Bähler, J., Shahrezaei, V., and Marguerat, S. (2019). Single-cell imaging and rna sequencing reveal patterns of gene expression heterogeneity during fission yeast growth and adaptation. *Nature microbiology*, **4**(3), 480–491.

[15] Sun, M. and Zhang, J. (2020). Allele-specific single-cell rna sequencing reveals different architectures of intrinsic and extrinsic gene expression noises. *Nucleic acids research*, **48**(2), 533–547.

[16] Sunnåker, M., Busetto, A. G., Numminen, E., Corander, J., Foll, M., and Dessimoz, C. (2013). Approximate bayesian computation. *PLOS Computational Biology*, **9**, e1002803.

[17] Tang, W., Bertaux, F., Thomas, P., Stefanelli, C., Saint, M., Marguerat, S., and Shahrezaei, V. (2019). bayNorm: Bayesian gene expression recovery, imputation and normalization for single-cell RNA-sequencing data. *Bioinformatics*, **36**(4), 1174–1181.

[18] Ximerakis, M., Lipnick, S. L., Innes, B. T., Simmons, S. K., Adiconis, X., Dionne, D., Mayweather, B. A., Nguyen, L., Niziolek, Z., Ozek, C., *et al.* (2019). Single-cell transcriptomic profiling of the aging mouse brain. *Nature neuroscience*, **22**(10), 1696–1708.

S2 Supplementary figures

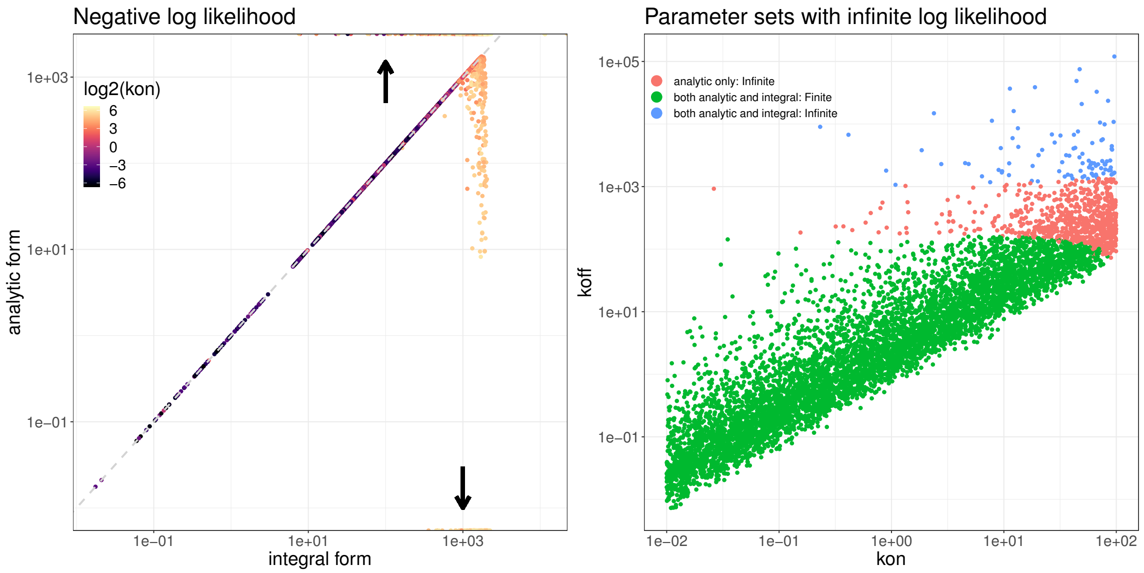

Figure S1: Two ways of calculating the negative log-likelihood. Using the simulated data and the corresponding ground truth parameters, the negative log-likelihoods of most genes are almost the same whether we compute them using the integral method or the analytic form given in (Eq. (1)). However, the analytic form is more likely to give an infinite outcome as the upper bound and 0 as the lower bound than the integral method. The arrows in the first panel highlight this property. The inconsistencies between the two approaches stem from parameter sets where the k_{on} is high.

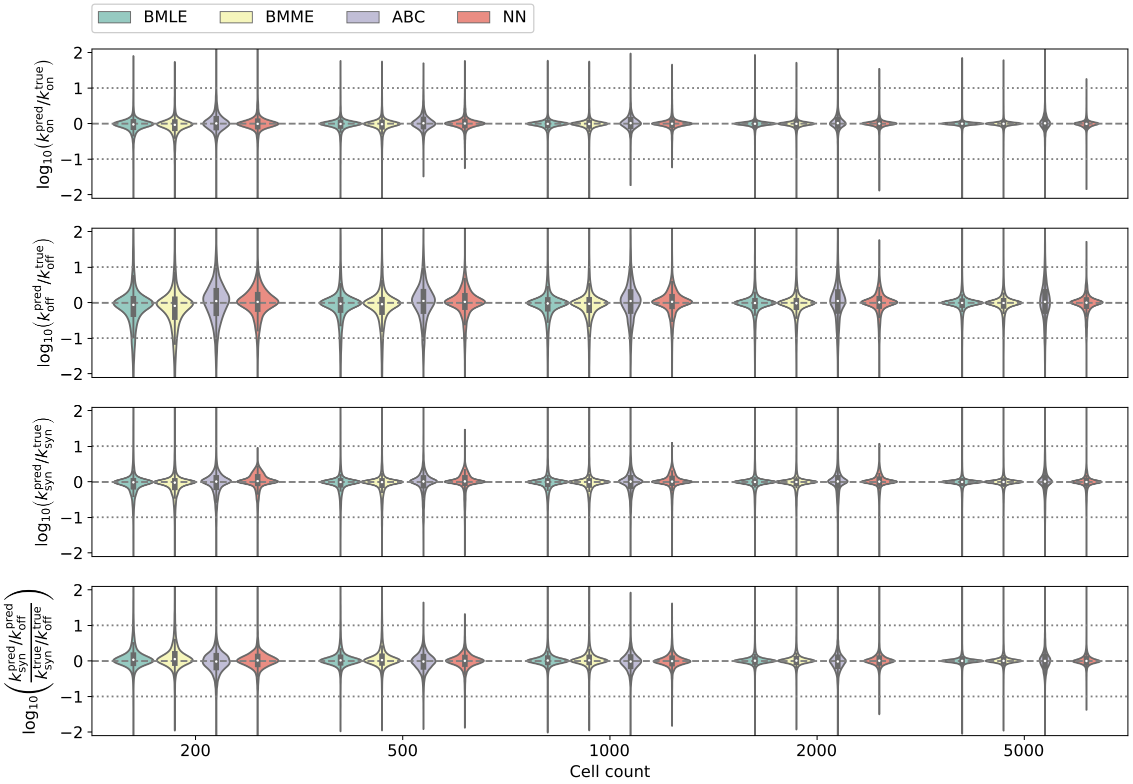

Figure S2: Comparison between different modelling approaches as a function of the number of cells at a fixed capture efficiency of 1.0 for allele-specific synthetic data. For each number of cells, the synthetic data set contains 1000 genes with 20 repetitions each.

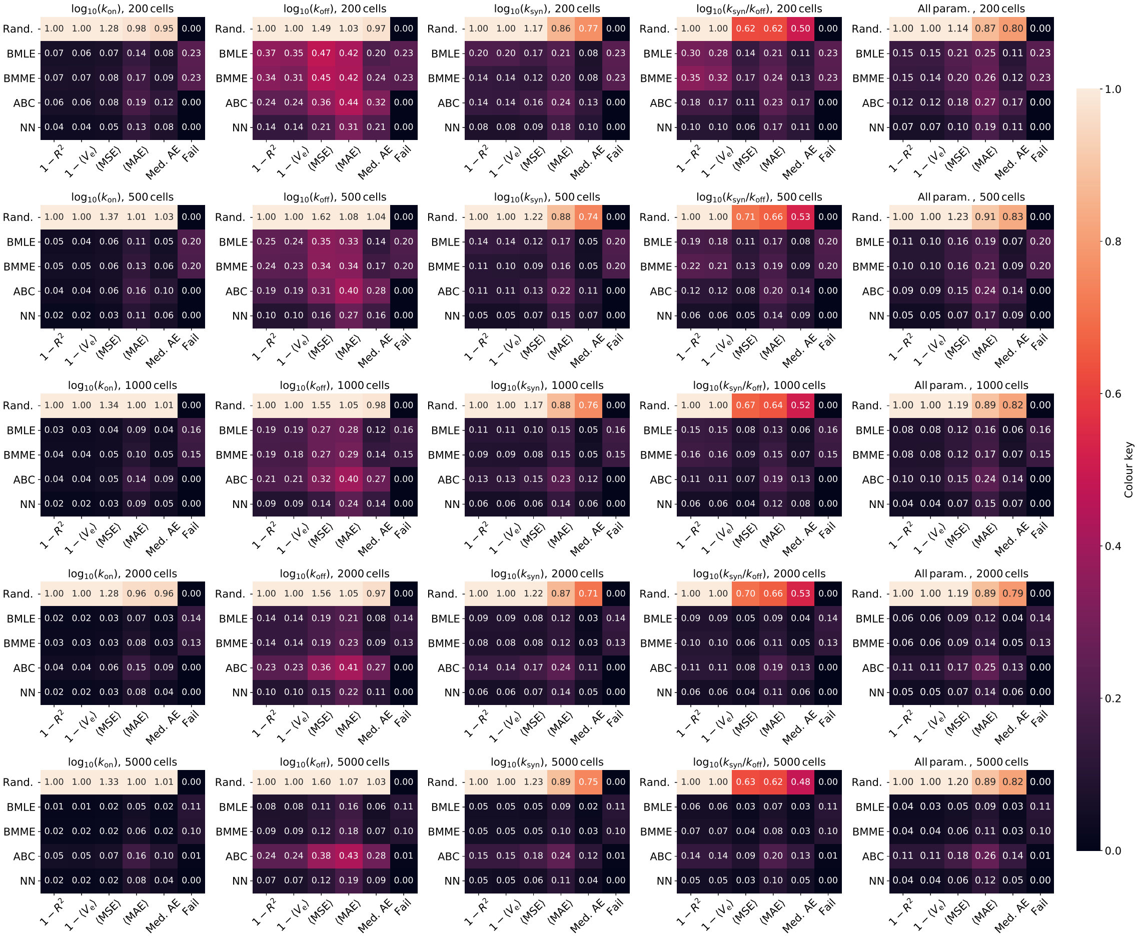

Figure S3: Performance metrics across methods and cell counts based on allele-specific synthetic data with a fixed capture efficiency of 1.0. For all metrics, lower numbers imply better performance. These metrics include the coefficient of determination (R^2), the explained variance (V_e), the mean squared error (MSE), the mean absolute error (MAE), the median absolute error (med. AE), and the failure rate, i.e. the fraction of test cases, for which the method is unable to provide parameter estimates. To put the scores into perspective, the upper row of each heat map includes the results (rand.) that are obtained when consistently guessing the parameters to take the mean value of the ground truth across all samples. For all approaches, we use a uniform prior for the logarithm of the Fano factor.

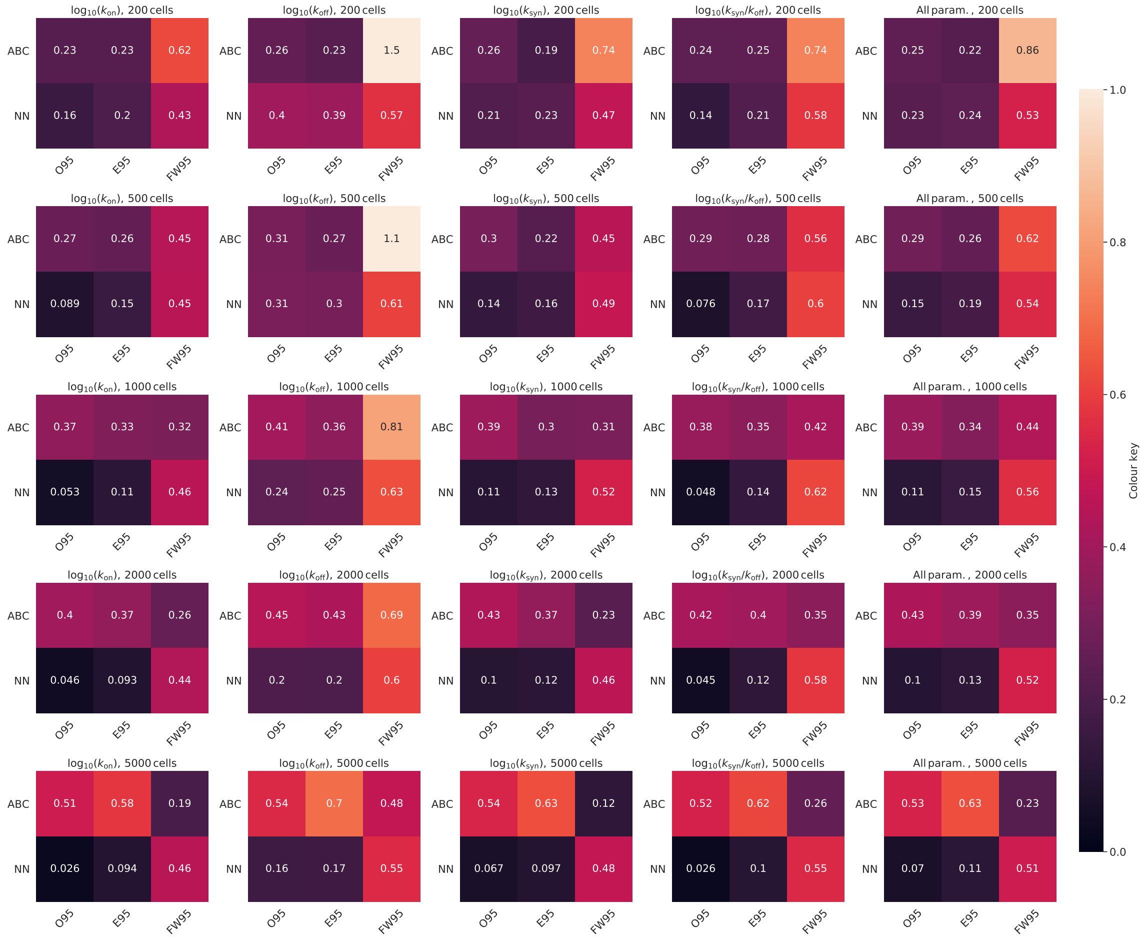

Figure S4: Additional performance metrics across methods and cell counts related to the predicted 95 % credibility intervals. The plot is based on allele-specific synthetic data with a fixed capture efficiency of 1.0. O95 denotes the fraction of the true parameter values that lie outside of the 95 % credibility intervals. FW95 denotes the width of the 95 % credibility intervals in logarithmic space, while E95 is the median absolute error of the predictions in units of FW95. A uniform prior was employed for the logarithm of the Fano factor.

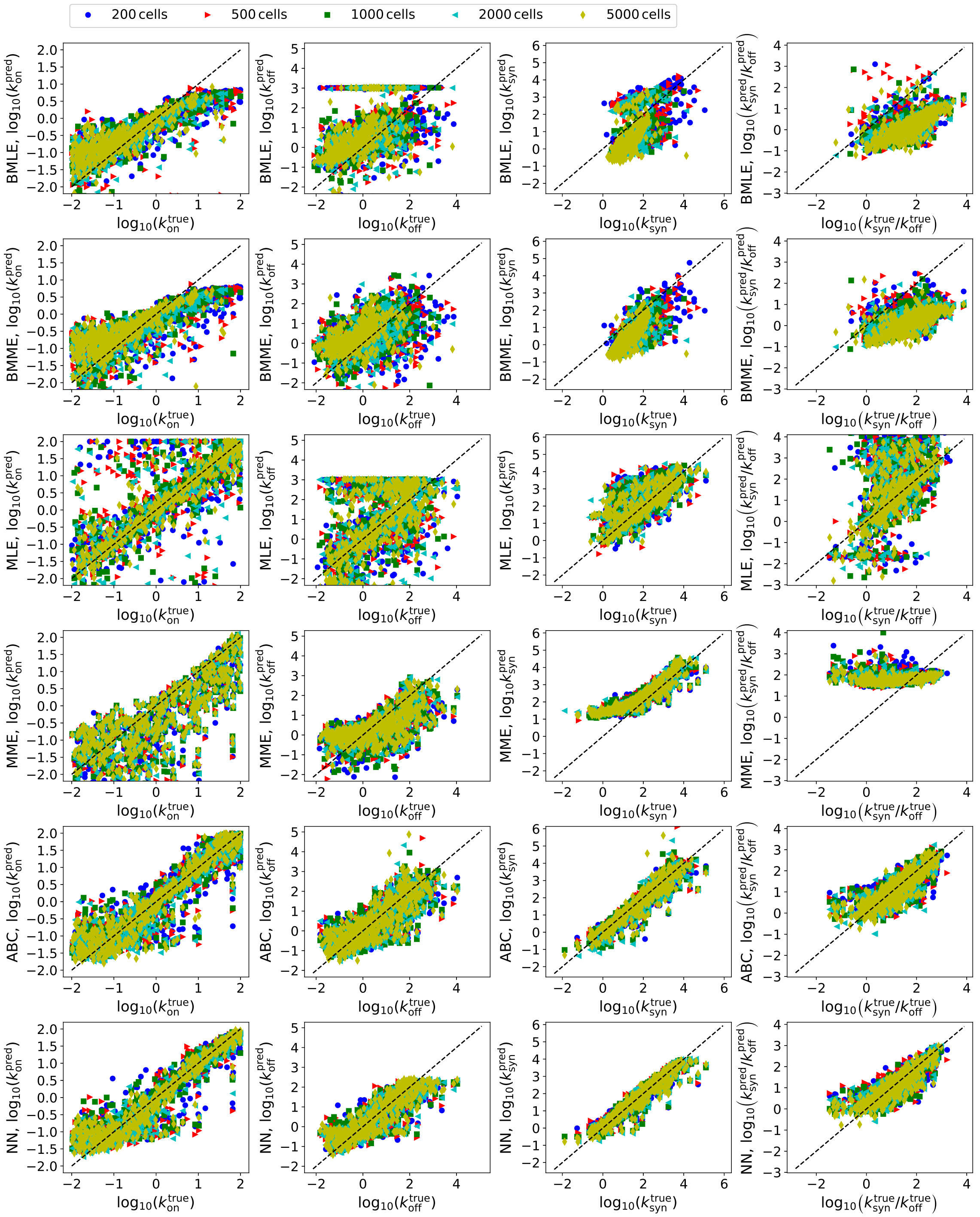

Figure S5: Scatter plot showing the predicted parameters as a function of the ground truth across different methods and cell counts. The plot includes 500 cells for each method and is based on allele-specific synthetic data.

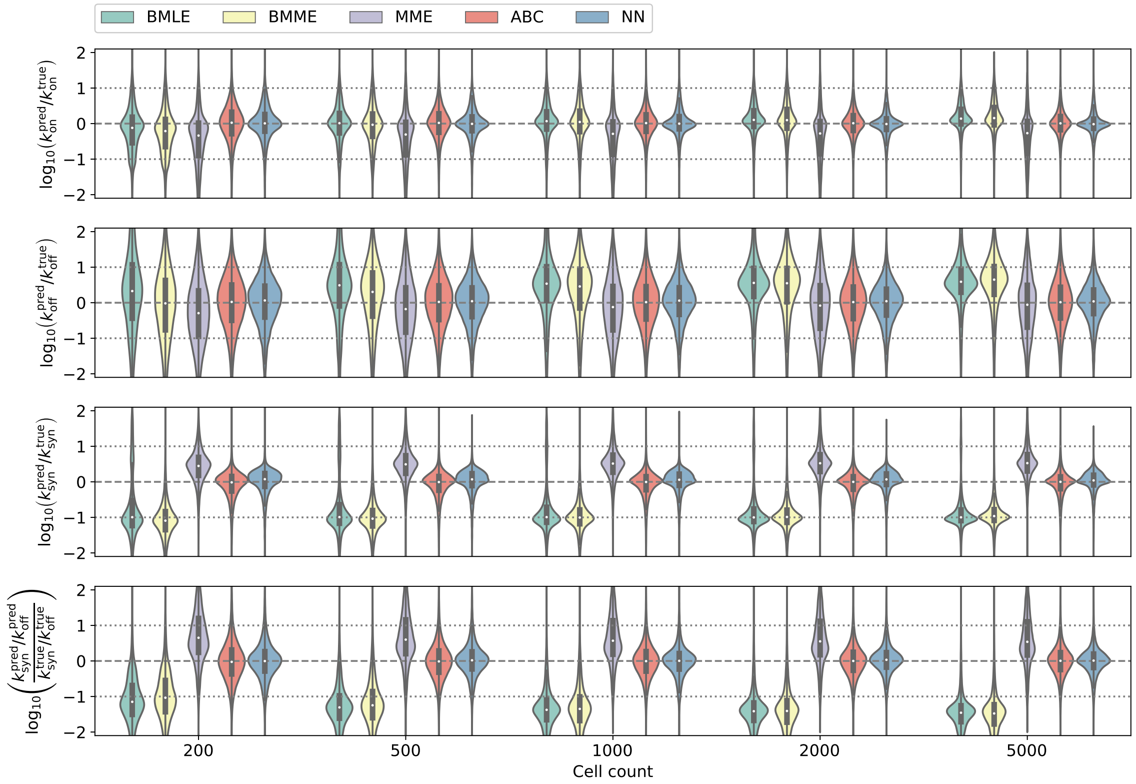

Figure S6: Comparison between different modelling approaches as a function of the number of cells for a varying capture efficiency with $\bar{\beta} = 0.06$ for allele-specific synthetic data. For each number of cells, the synthetic data set contains 7000 genes with 20 repetitions each.

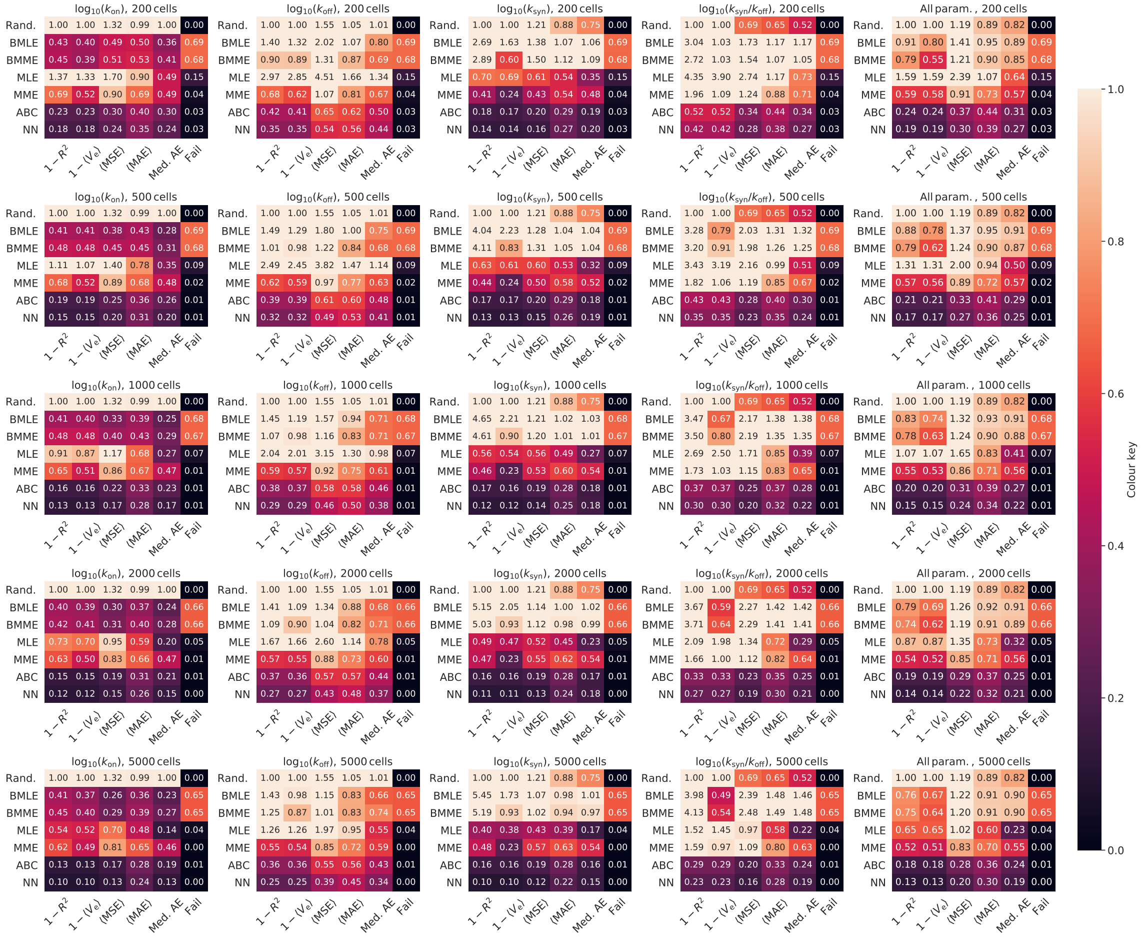

Figure S7: Performance metrics across methods and cell counts based on allele-specific synthetic data with a varying capture efficiency ($\bar{\beta} = 0.06$). For more information, see the caption of Fig. S3.

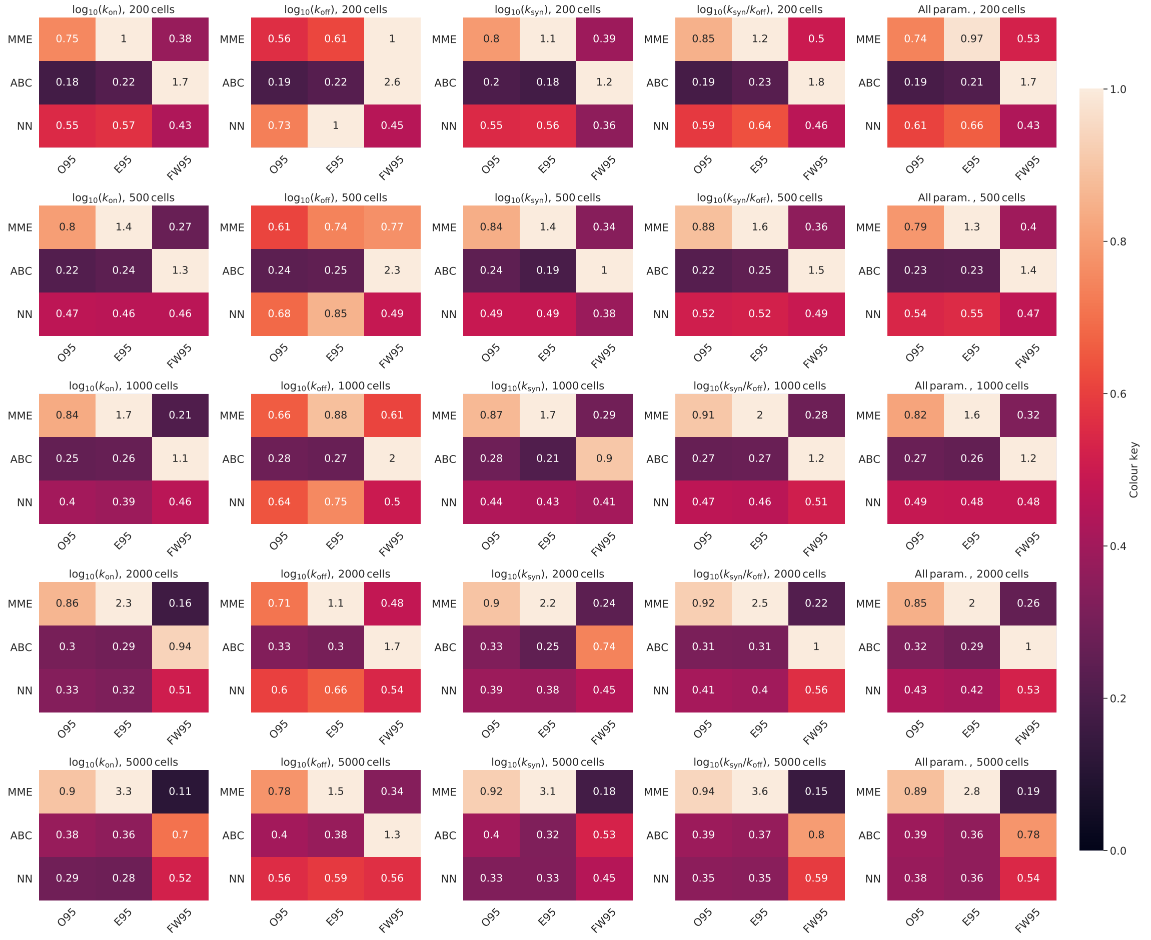

Figure S8: Additional performance metrics across methods and cell counts related to the predicted 95 % credibility intervals. The plot is based on allele-specific synthetic data with a varying capture efficiency ($\bar{\beta} = 0.06$). For further details see Fig. S4.

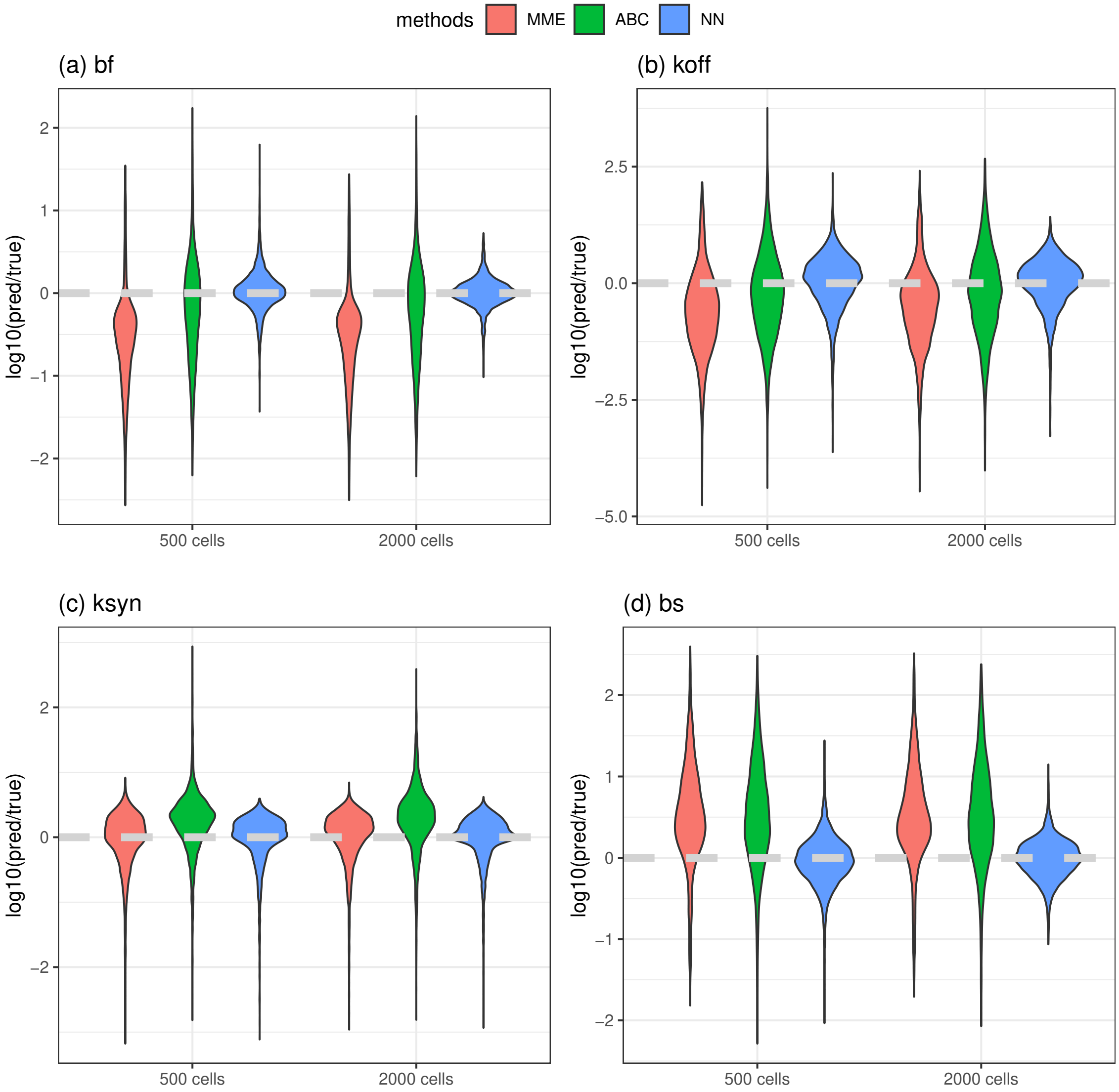

Figure S9: Comparison of inference methods. Based on non-allele specific simulated data with the mean capture efficiency set to be 0.06. The ratio between estimates and ground truth of burst frequency **(a)**; koff **(b)**; ksyn **(c)** and burst size **(d)** are shown.

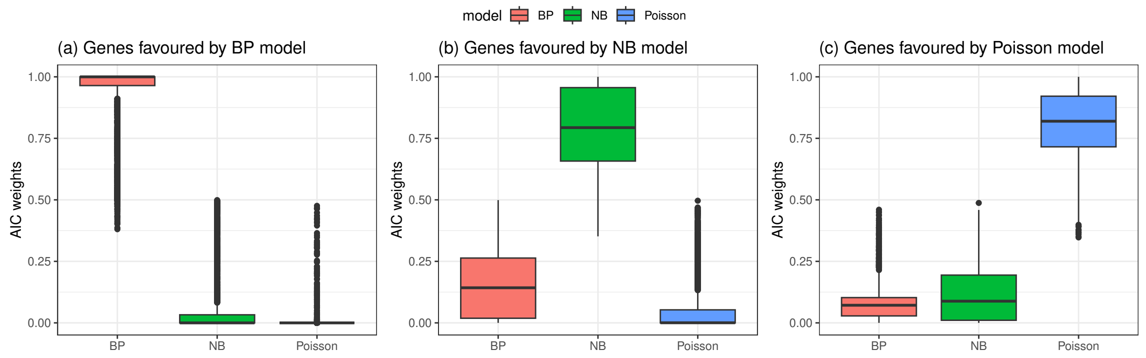

Figure S10: Figure related to Fig. 3: Box plots of AIC weights of genes favored by different models: (a) BP; (b) NB and (c) Poisson.

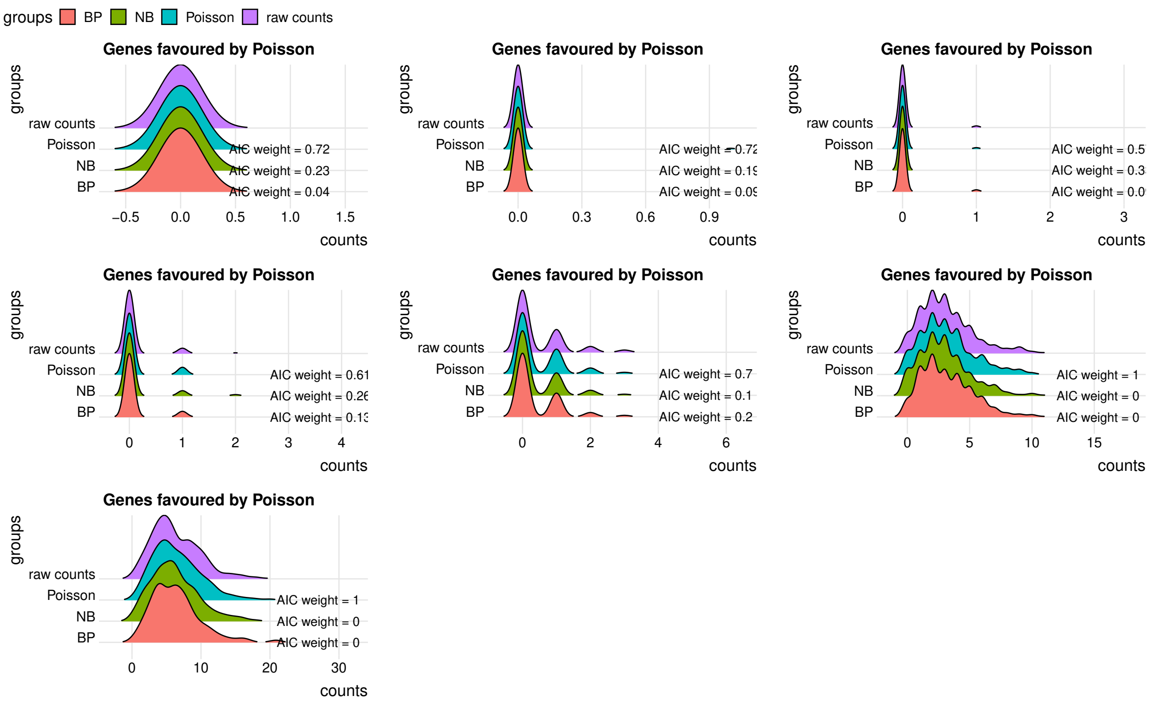

Figure S11: Figure related to Fig. 3. Each panel represents an example gene favoured by Poisson model. Distribution of raw counts simulated from the BP model and the likelihood of the different models after binomial dowsampling with cell specific capture efficiencies are shown. Selected genes were ordered according to their mean expression from low to high (from left to right, from top to bottom).

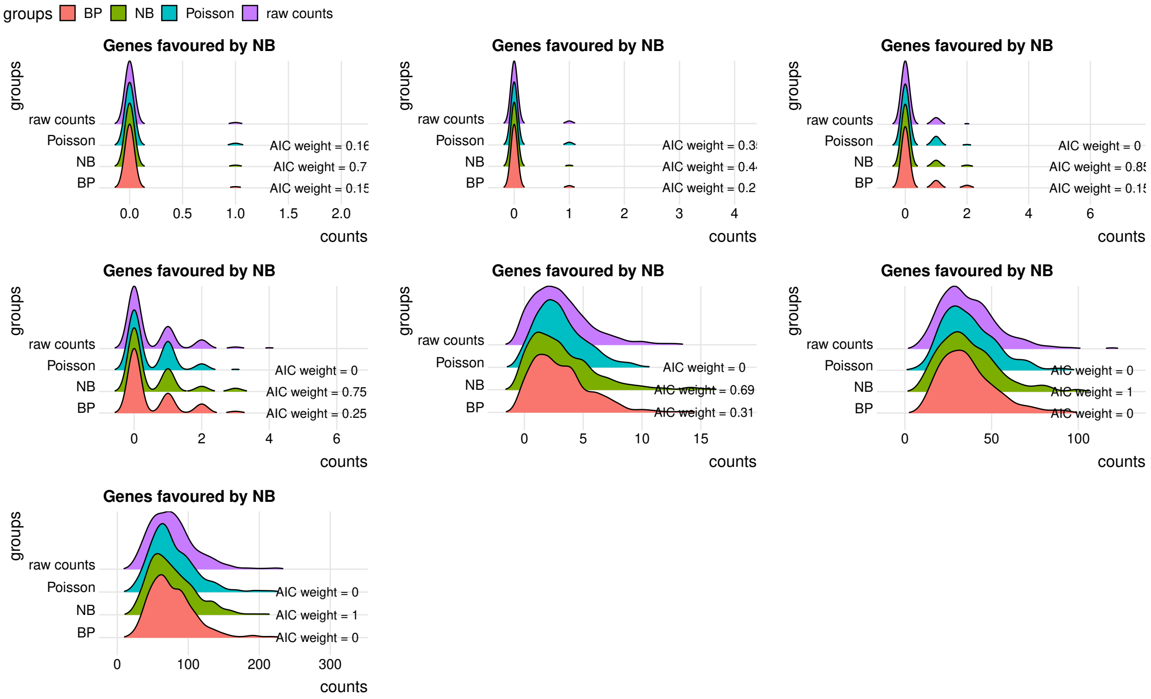

Figure S12: Figure related to Fig. 3. Each panel represents an example gene favoured by NB model. Distribution of raw counts simulated from the BP model and the likelihood of the different models after binomial dowsampling with cell specific capture efficiencies are shown. Selected genes were ordered according to their mean expression from low to high (from left to right, from top to bottom).

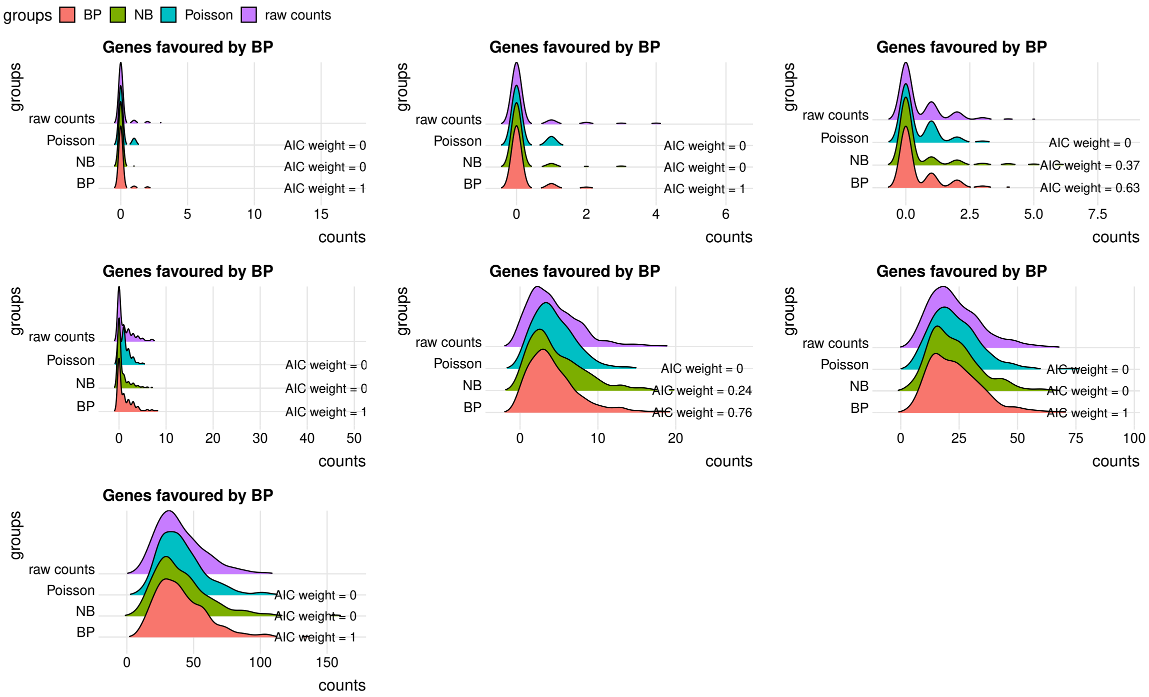

Figure S13: Figure related to Fig. 3. Each panel represents an example gene favoured by BP model. Distribution of raw counts simulated from the BP model and the likelihood of the different models after binomial dowsampling with cell specific capture efficiencies are shown. Selected genes were ordered according to their mean expression from low to high (from left to right, from top to bottom).

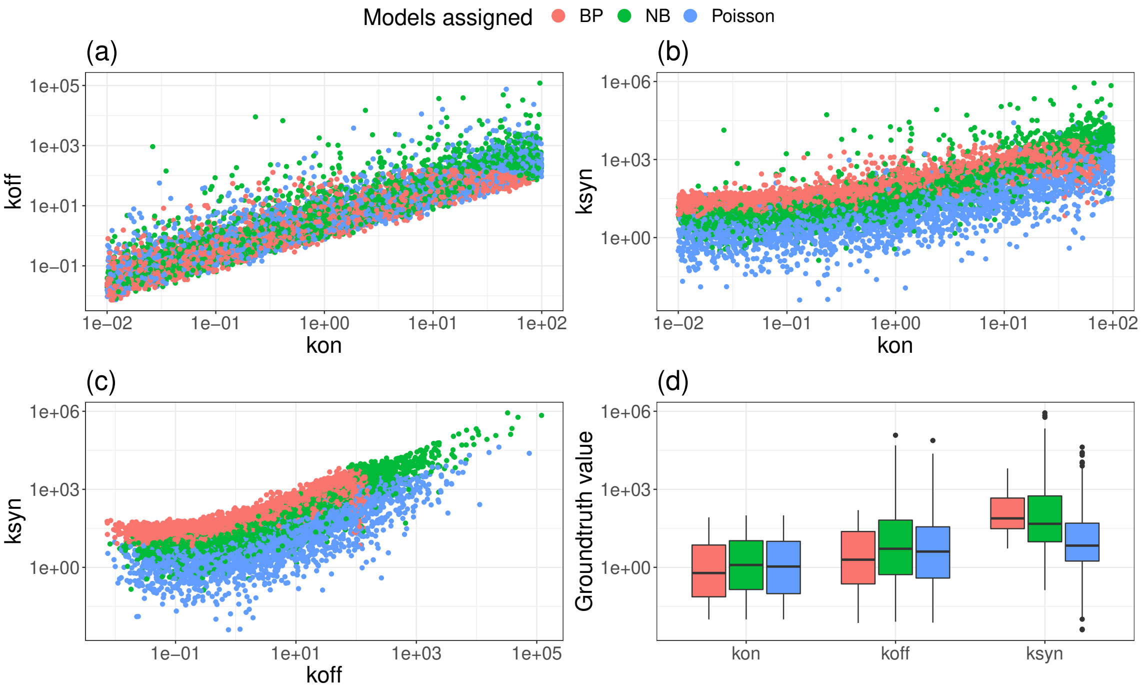

Figure S14: Low k_{syn} lead to wrong identification. **(a-c)**: each dot represents one gene. Model selection of BP (Beta-Poisson), NB (negative binomial) and Poisson distributions based on the lowest AIC. **(d)** Impact of the magnitude of the kinetic parameters on the model selection.

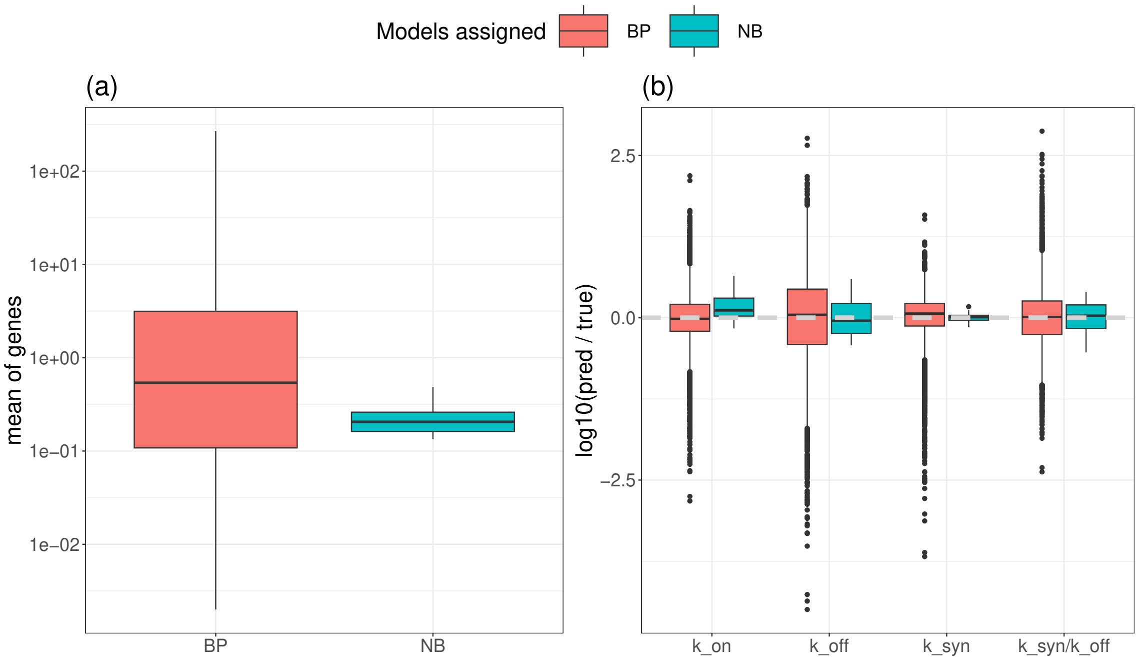

Figure S15: Figure related to Fig. 3: Instead of AIC, we applied WAIC for model selection for every gene. None of the genes were found to be generated from the Poisson model. In agreement with Fig. 3 which is based on AIC, this analysis again shows that genes with low counts are assigned to simpler models. **(a)** Based on synthetic data generated by the Beta-Poisson model, genes were labelled to be from one of the three models according to their WAIC value. The mean counts for genes assigned to each model are shown. **(b)** The ratios between inferred and true parameter values in each group of genes are shown. Estimates from genes which are assigned to BP correctly are closer to the ground truth values.

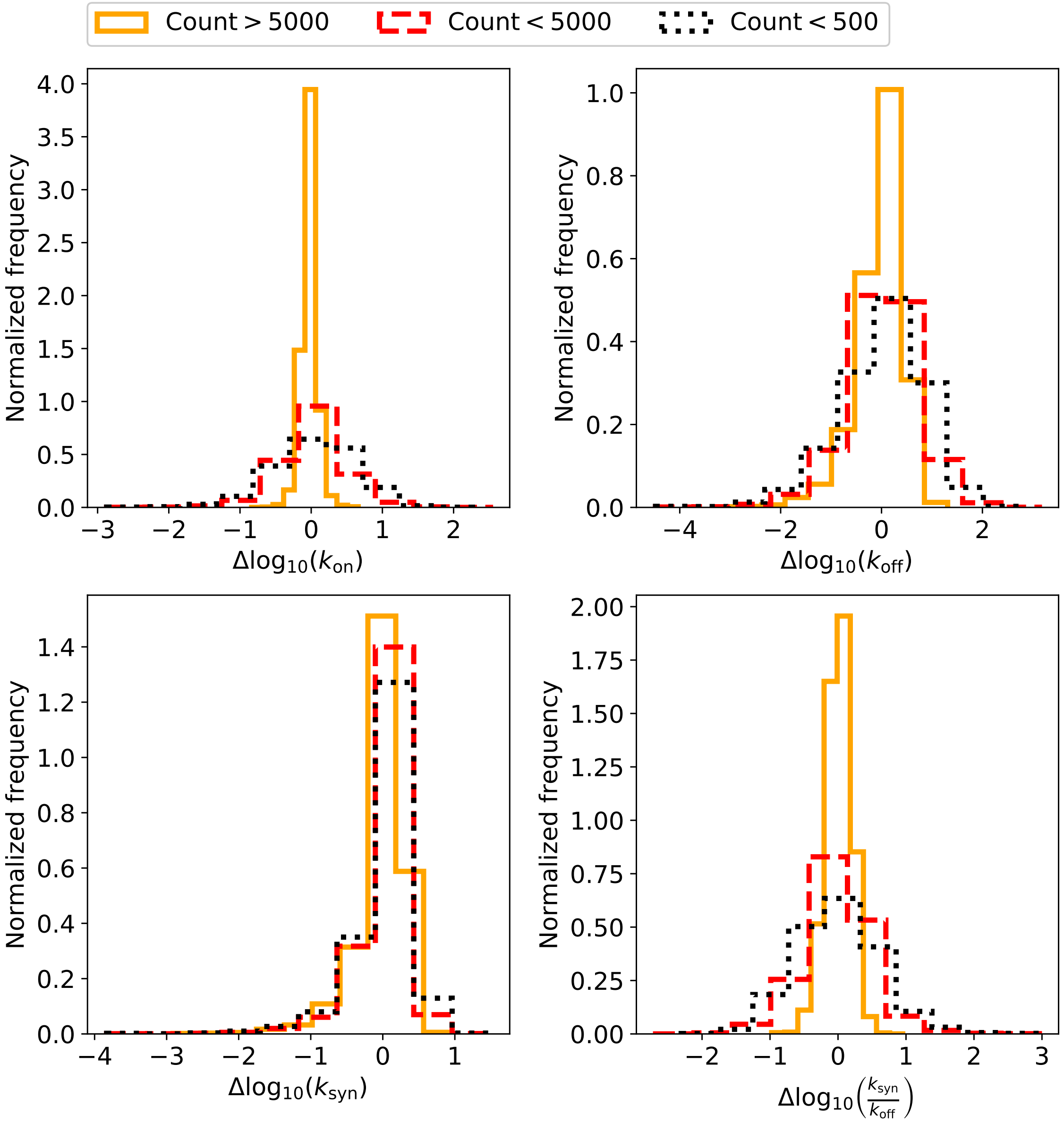

Figure S16: Histogram summarizing the errors in the predicted kinematic parameters for our synthetic data (Fig. S5) based on the NN for 5000 cells. The genes have been categorized based on their expression. We thus distinguish between those genes that have fewer than 500 counts, those that have fewer than 5000 counts and those that have more than 5000 counts across all 5000 cells. As can be seen from the figure, lowly expressed genes generally lead to higher errors.

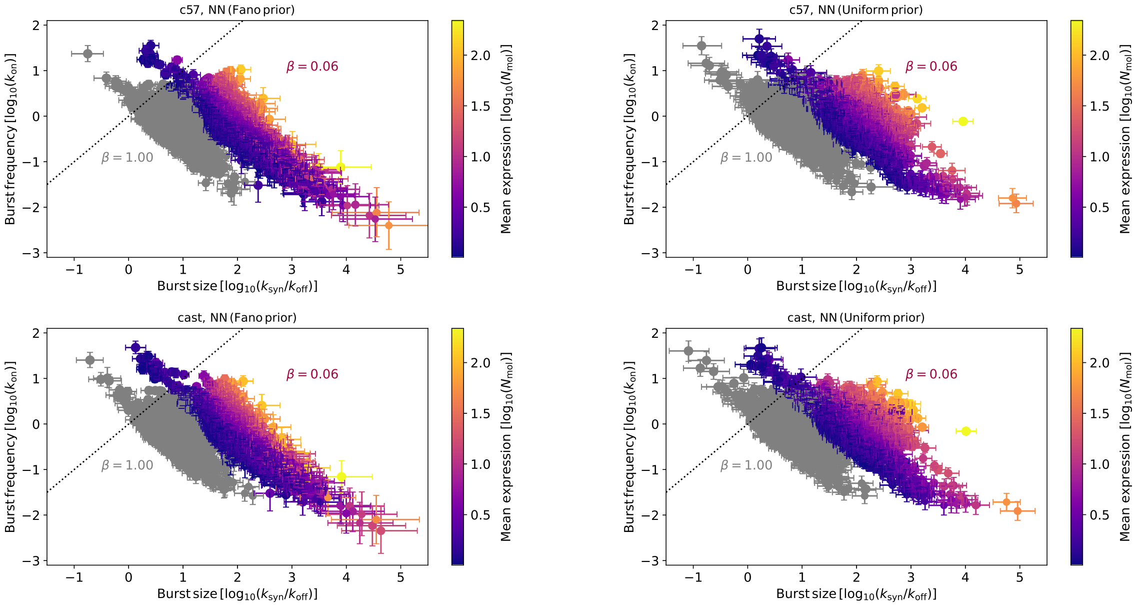

Figure S17: Predicted burst frequency and burst size for each allele (CAST/EiJ × C57BL/6J) based on data from [11]. The burst kinetics were inferred using the neural network. During the training, we either imposed a uniform prior for the logarithm of the Fano factor (left-hand side) or directly for the logarithm of k_{syn} (right-hand side). Two scenarios for the capture efficiency (β) were considered: A fixed capture efficiency of $\beta = 1.0$ (grey markers) and a varying capture efficiency with $\bar{\beta} = 0.06$. The error bars signify 68 % credibility intervals.

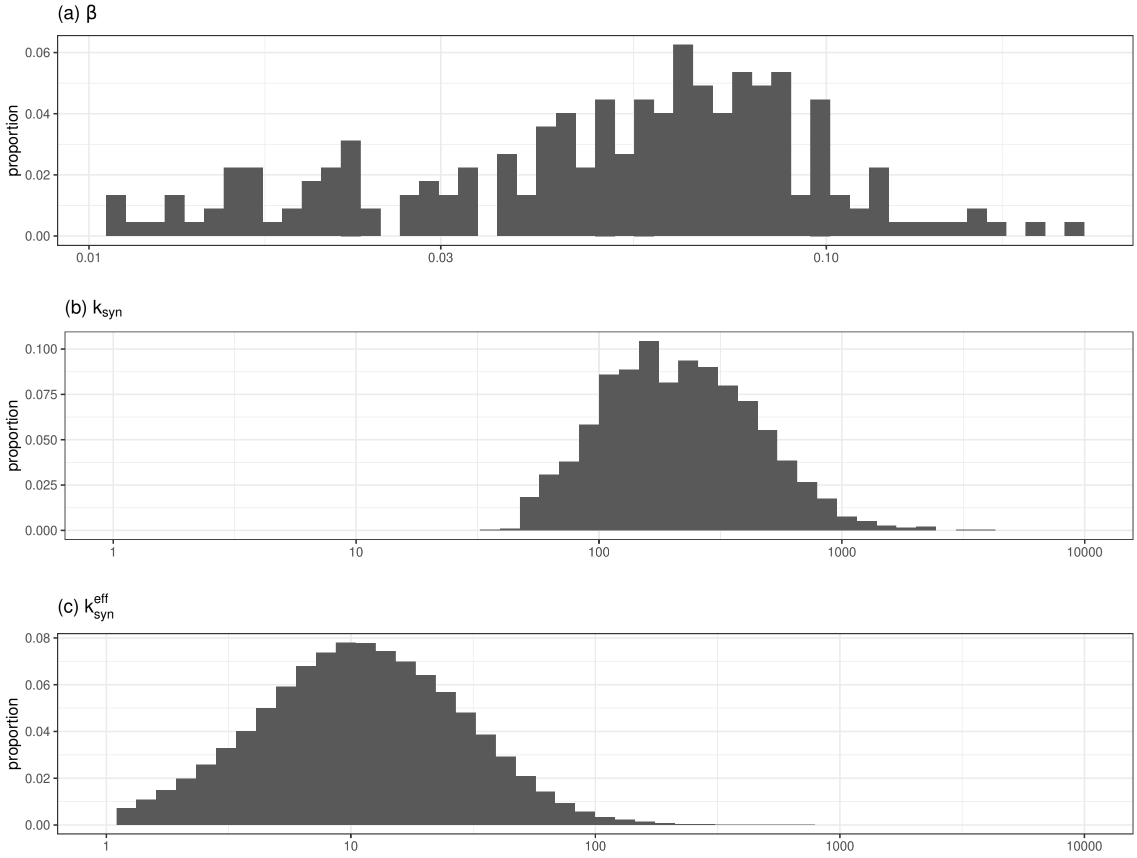

Figure S18: Inferred results for Allele c57. **(a)**: estimated $\hat{\beta}_j = s_j \beta_j$ from c57 data; **(b)**: estimated k_{syn} from c57 data across all genes. **(c)**: distribution of k_{syn}^{eff} across all cells and genes (product of cell specific $\hat{\beta}$ and gene specific k_{syn}).

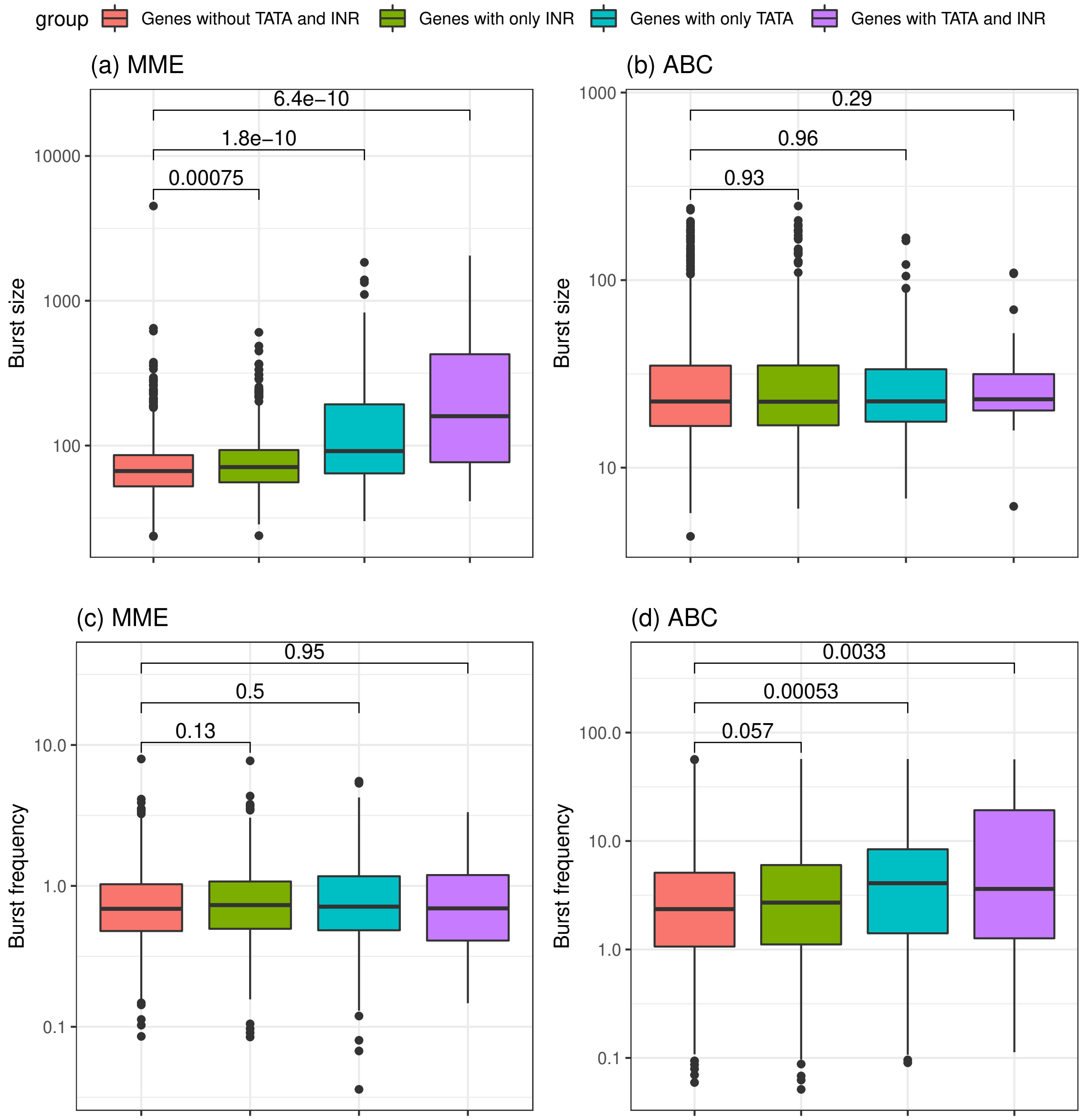

Figure S19: Figure related to Fig. 4. Allele c57. **(a-b)**: burst size inferred from MME and ABC; **(c-d)**: burst frequency inferred from MME and ABC. The P-values of the Wilcox test between groups are shown.

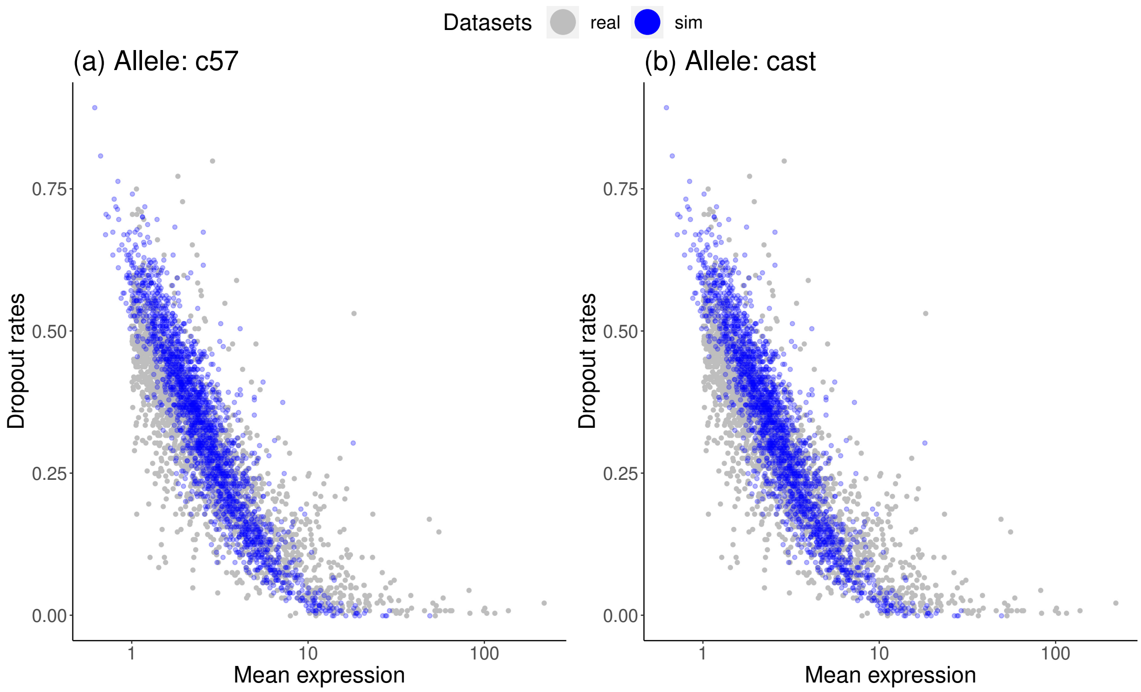

Figure S20: Comparison between real and simulated data in terms of the dropout-mean relationship. Simulated data were generated based on parameters estimated using the NN method. Kinetic parameters were inferred based on data from **(a)** allele c57 and **(b)** allele cast, respectively.

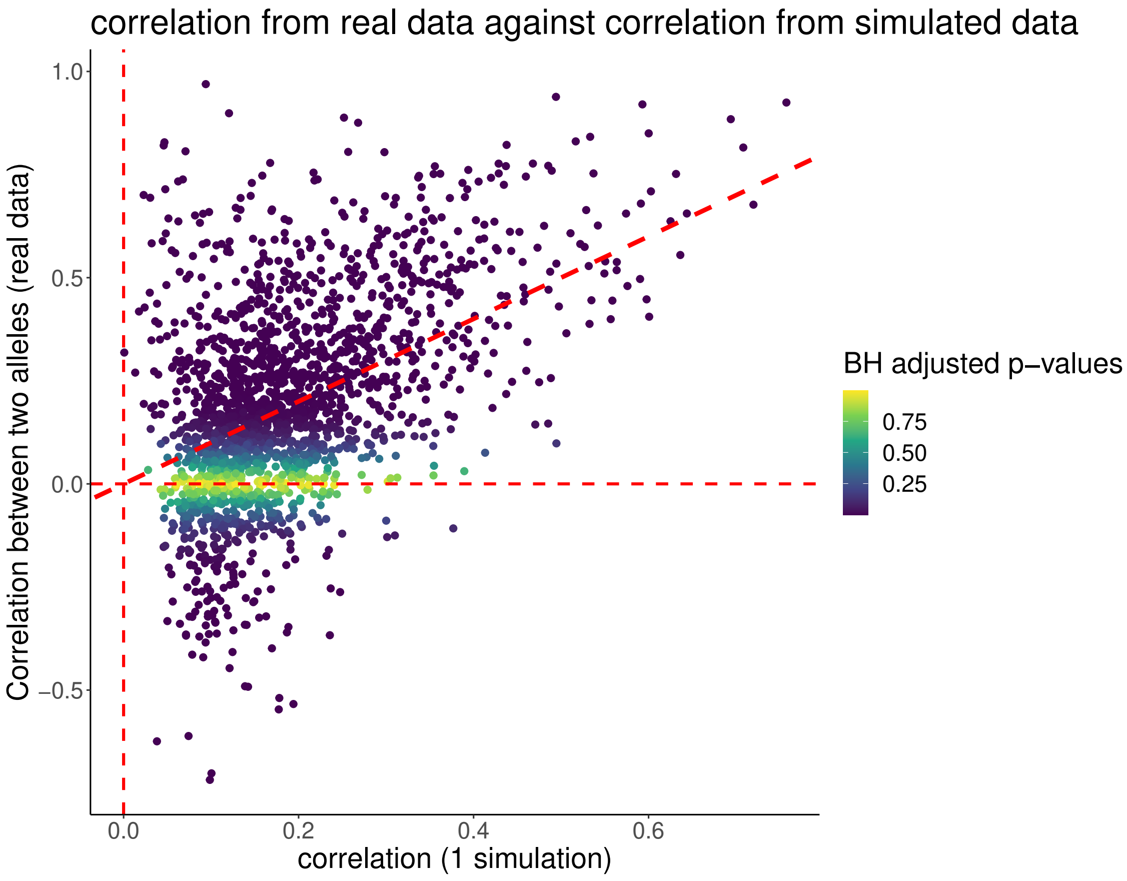

Figure S21: Kinetic parameters inferred from allele-specific data Larsson *et al.* [11]. Each point represents the Spearman correlation between two alleles for one gene. Dots are coloured according to the adjusted P-values calculated using the real data. The three dashed lines are a diagonal line ($x = y$), a vertical line at $x = 0$ and a horizontal line at $y = 0$, respectively.

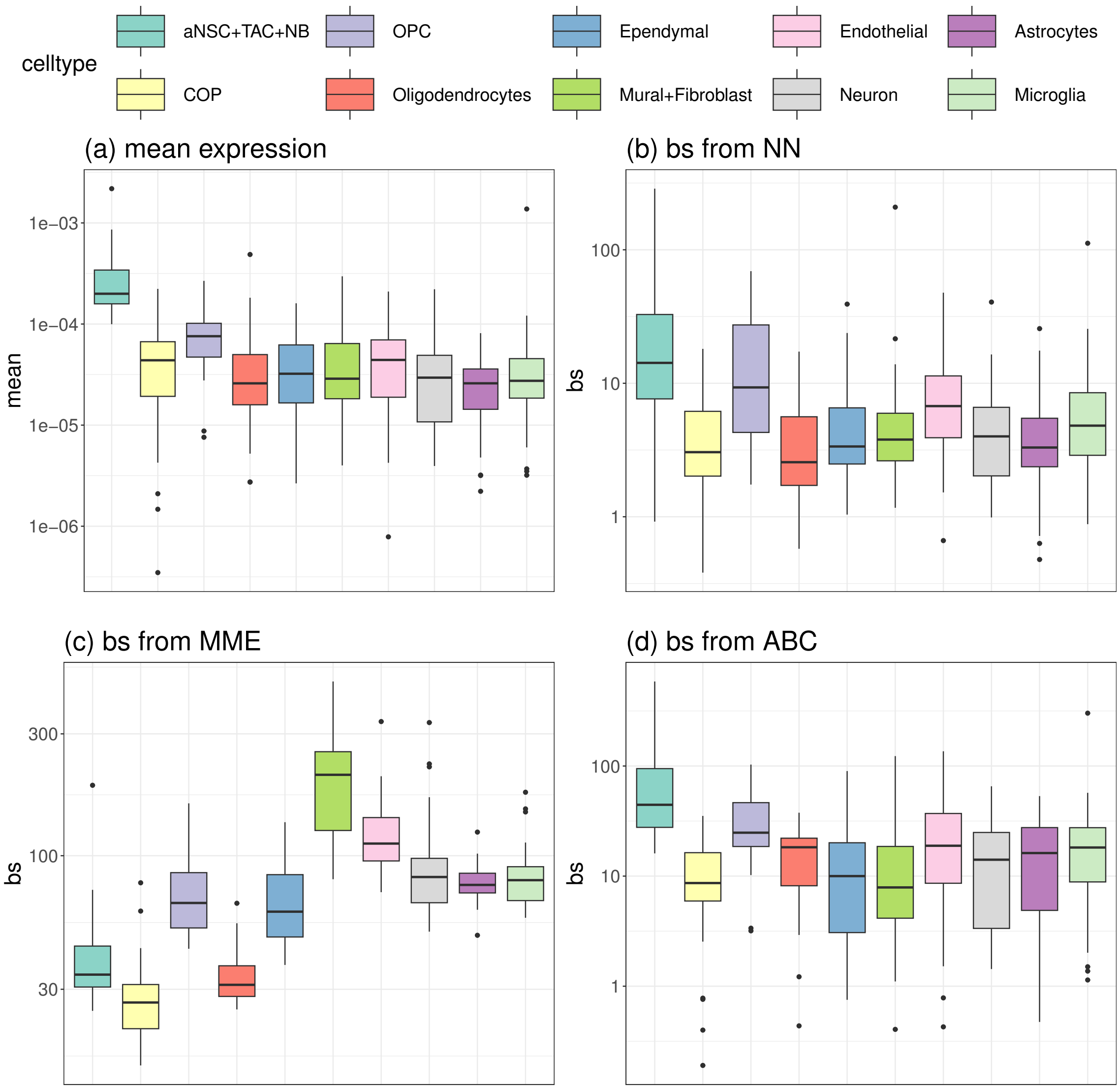

Figure S22: Figure related to Fig. 5. **(a)** Mean expression, calculated after the total count was normalized; Burst size estimated using NN **(b)**, MME **(c)** and ABC **(d)**. Here, we use the aNSC marker genes reported in Mizrak *et al.* [12].

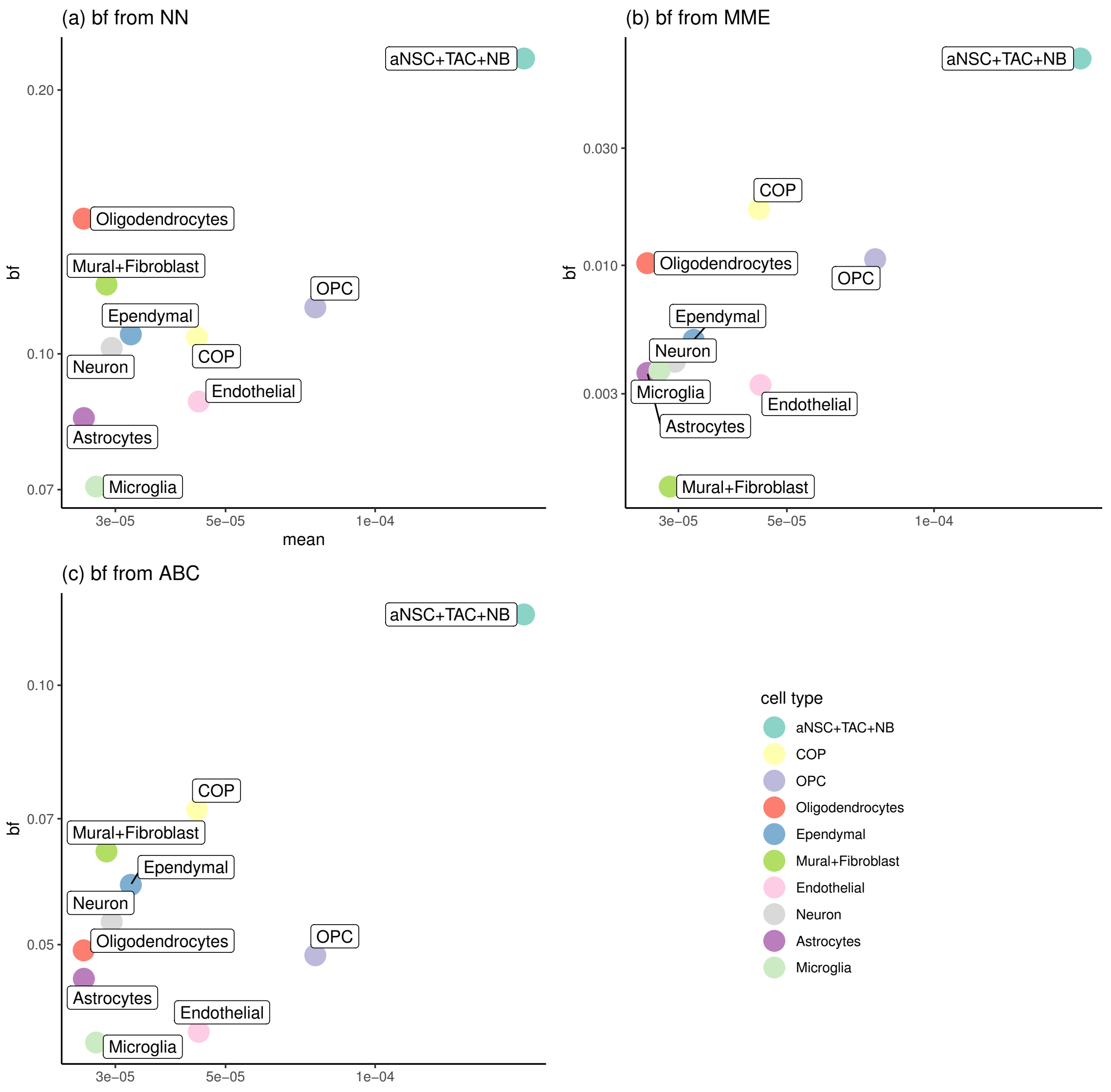

Figure S23: Figure related to Fig. 5. The medians of the box plot in Fig. 5 are shown on the x-axis. **(a)** Mean expression, calculated after total count normalized; Burst frequency estimated using , MME **(b)** and ABC **(c)**. Here, we use the aNSC marker genes reported in Mizrak *et al.* [12].

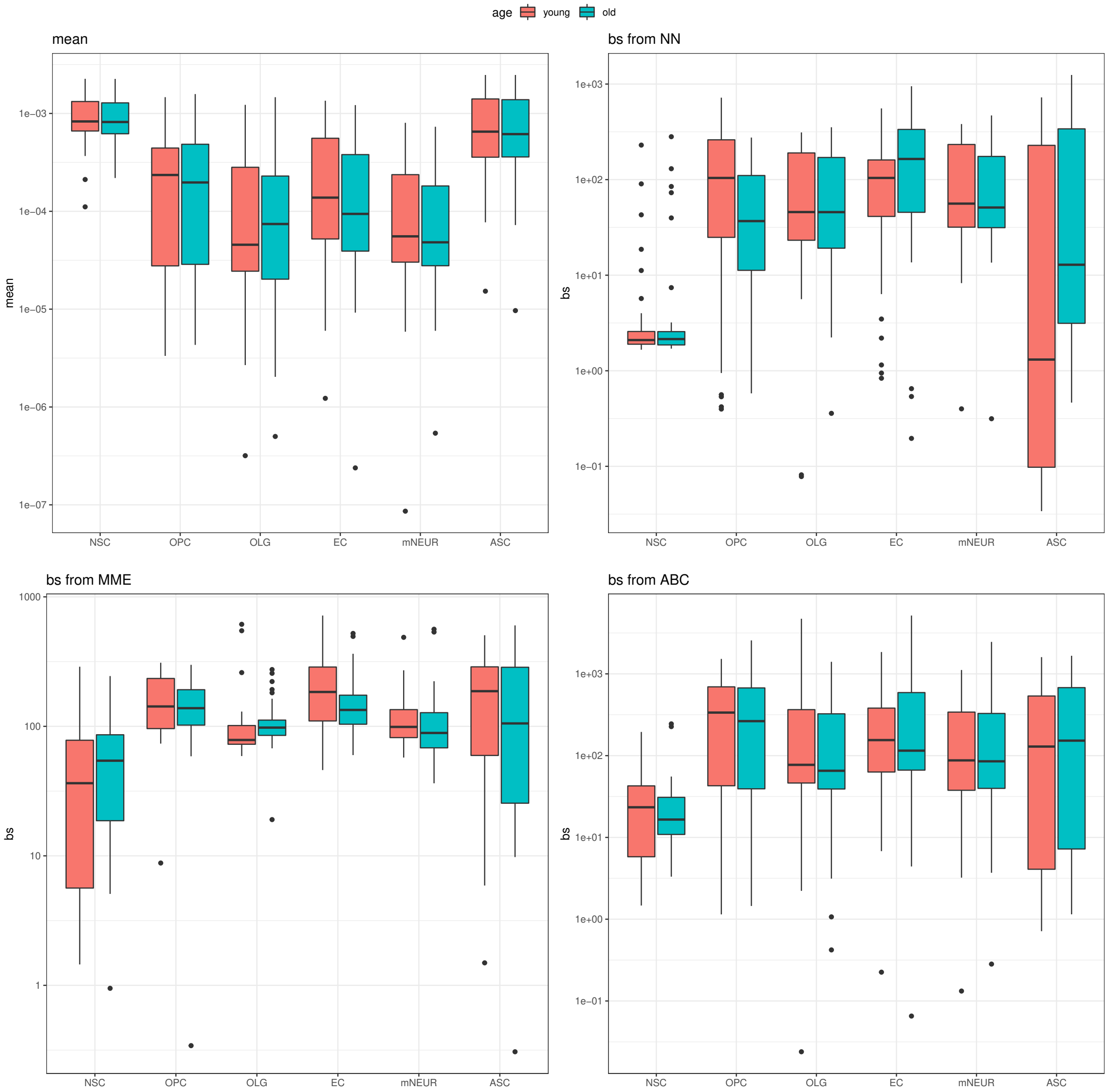

Figure S24: Figure related to Fig. 6. **(a)** Mean expression, calculated after total count normalized; Burst frequency estimated using NN **(b)**, MME **(c)** and ABC **(d)**. Here, we use the NSC marker genes reported in Ximerakis *et al.* [18].

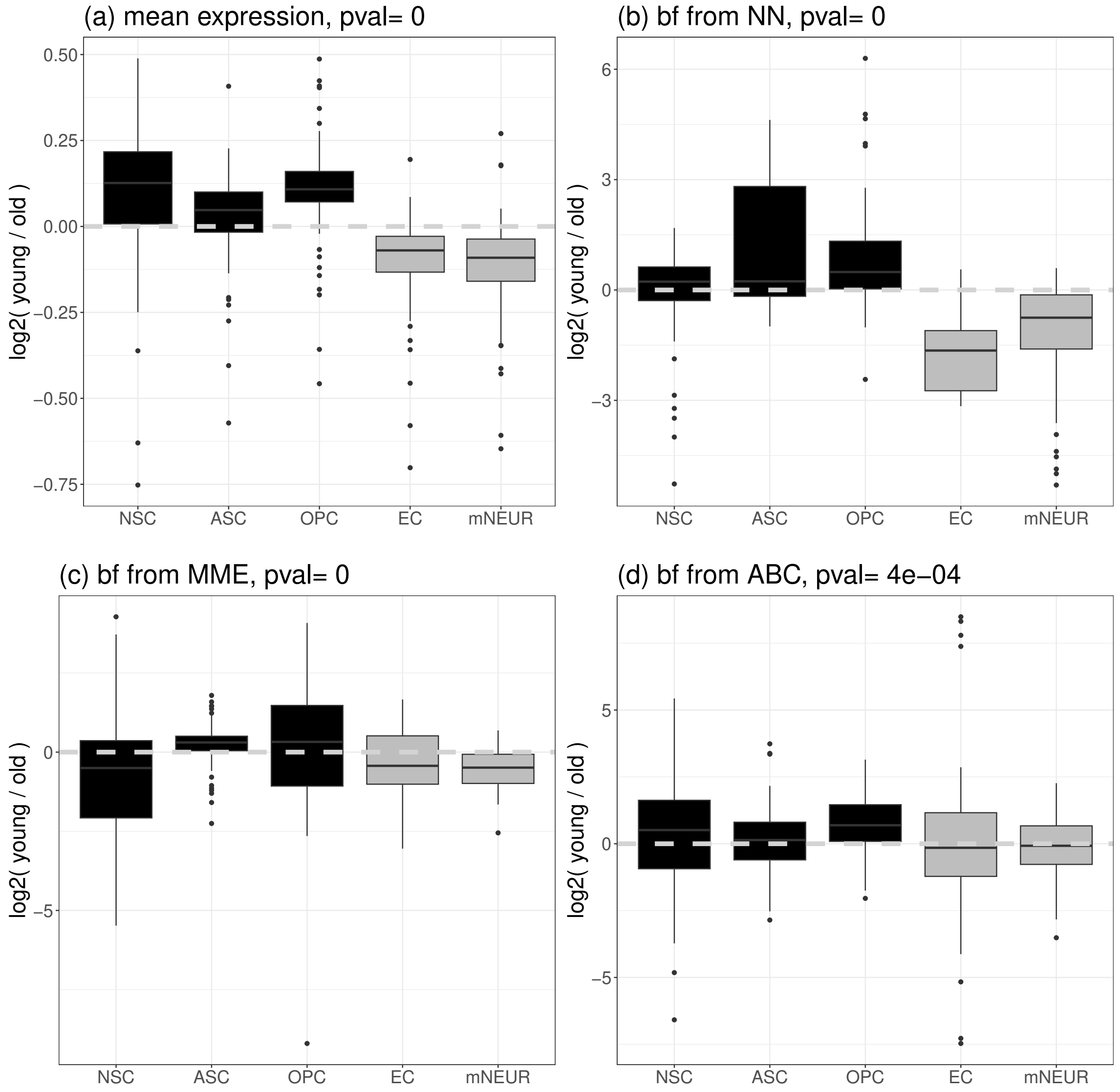

Figure S25: The ratio of burst frequency of genes encoding ribosomal subunits from young and old mice within each cell type. The P-value is based on the Wilcox test between NSC/ASC/OPC and mature cells. **(a)** Box plots of the ratio of the mean expression of genes encoding ribosomal genes. Mean expressions were calculated based on total count normalized data; Box plots of the ratio of inferred burst frequencies using NN **(b)**, MME **(c)** and ABC **(d)** inference approach.

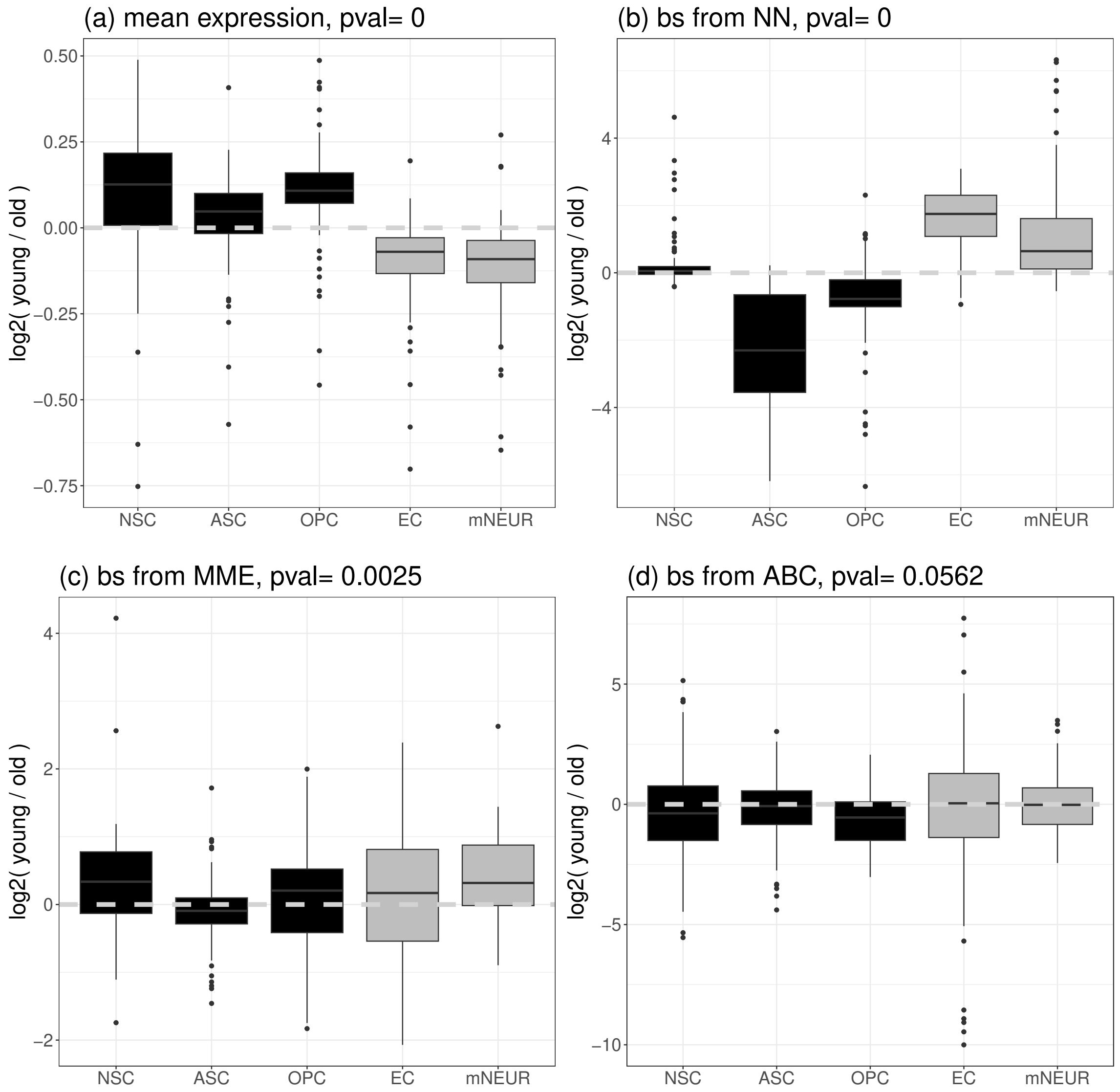

Figure S26: Figure related to Fig. S25. The ratio of the burst size of genes encoding ribosomal subunits within each cell type from young and old mice, respectively. The P-value is based on the Wilcox test between NSC/ASC/OPC and mature cells. **(a)** Mean expression, calculated after the total count was normalized; Burst size estimated using NN **(b)**, MME **(c)** and ABC **(d)**.
